# Characterization of the microbiome and volatile compounds in anal gland secretions from domestic cats (*Felis catus*) using metagenomics and metabolomics

**DOI:** 10.1038/s41598-023-45997-1

**Published:** 2023-11-08

**Authors:** Connie A. Rojas, Stanley L. Marks, Eva Borras, Hira Lesea, Mitchell M. McCartney, David A. Coil, Cristina E. Davis, Jonathan A. Eisen

**Affiliations:** 1 Genome Center, University of California–Davis, Davis, CA USA; 2 Department of Evolution and Ecology, University of California–Davis, Davis, CA USA; 3 Department of Medicine and Epidemiology, University of California–Davis, School of Veterinary Medicine, Davis, CA USA; 4 Department of Mechanical and Aerospace Engineering, University of California–Davis, Davis, CA USA; 5 UC Davis Lung Center, University of California–Davis, Davis, CA USA; 6 Department of Microbiology and Molecular Genetics, University of California–Davis, Davis, CA USA; 7 VA Northern California Health Care System, Mather, CA USA; 8 Department of Medical Microbiology and Immunology, University of California-Davis, Davis, CA, USA

**Keywords:** Zoology, Animal behaviour, Ecology, Behavioural ecology, Community ecology, Microbial ecology, Microbiology, Bacteria, Microbial communities

## Abstract

Many mammals rely on volatile organic chemical compounds (VOCs) produced by bacteria for their communication and behavior, though little is known about the exact molecular mechanisms or bacterial species that are responsible. We used metagenomic sequencing, mass-spectrometry based metabolomics, and culturing to profile the microbial and volatile chemical constituents of anal gland secretions in twenty-three domestic cats (*Felis catus*), in attempts to identify organisms potentially involved in host odor production. We found that the anal gland microbiome was dominated by bacteria in the genera *Corynebacterium*, *Bacteroides*, *Proteus*, *Lactobacillus*, and *Streptococcus*, and showed striking variation among individual cats. Microbiome profiles also varied with host age and obesity. Metabolites such as fatty-acids, ketones, aldehydes and alcohols were detected in glandular secretions. Overall, microbiome and metabolome profiles were modestly correlated (r = 0.17), indicating that a relationship exists between the bacteria in the gland and the metabolites produced in the gland. Functional analyses revealed the presence of genes predicted to code for enzymes involved in VOC metabolism such as dehydrogenases, reductases, and decarboxylases. From metagenomic data, we generated 85 high-quality metagenome assembled genomes (MAGs). Of importance were four MAGs classified as *Corynebacterium frankenforstense*, *Proteus mirabilis*, *Lactobacillus johnsonii*, and *Bacteroides fragilis*. They represent strong candidates for further investigation of the mechanisms of volatile synthesis and scent production in the mammalian anal gland.

## Introduction

Many animals communicate using chemical cues^1–4^. These chemical compounds include volatile organic compounds (VOCs) found in feces, urine, saliva, and glandular secretions^1,5–9^ that can assist with kin recognition^10–12^, territory defense^13,14^, reproductive advertisement^15–18^, and can even deter predators^19,20^ or reduce the spread of parasites^21^. Glandular secretions in particular can encode information about the individual identity^22^, age^23^, sex^24,25^, reproductive status^15,26^, social status^27,28^ and social group^29,30^ of the sender. While mammalian hosts have some proteins that can bind or synthesize VOCs^31–33^, the majority of volatiles found in scent glands are actually produced by fermentative bacteria that reside within the gland itself. Several studies have examined the scent gland microbiomes and volatiles from a variety of species^25,30,34–36^, but few have linked specific bacterial taxa to the in vitro production of volatiles or to specific microbial gene pathways^34,35^.

The major constituents of mammalian odor are aldehydes, amides, alkanes, alcohols, hydrocarbons, fatty acids, esters, ketones, phenols, squalenes, and steroids^37^. Regarding the bacterial constituents, in the giant panda^35^, the most abundant bacterial genera of the anogenital gland—a cutaneous gland in the anogenital region—are *Corynebacterium*, *Pseudomonas*, *Porphyromonas*, *Psychrobacter*, and *Anaerococcus*. The anal glands of domestic dogs—situated between the internal and external anal sphincter muscles—mainly contain species from the bacterial genera *Enterococcus*, *Bacteroides*, and *Proteus *^38^. In wild spotted hyenas, anal glands are primarily populated by *Anaerococcus*, *Corynebacterium*, *Eubacterium*, *Porphyromonas*, and *Propionibacterium *^39^.

A few experimental studies have found more direct links between specific bacterial species and the production of odor compounds in scent-producing glands. In dark-eyed juncos, and hoopoes, treating glandular secretions with antibiotics changes the composition of the microbial community and inhibits the production of volatile compounds^40,41^. Most recently, a metagenomic survey of giant panda anogenital gland secretions identified several metabolic pathways likely involved in the production of VOCs^35^. Among those pathways were fatty acid biosynthesis, the synthesis and degradation of ketone bodies, steroid biosynthesis, the biosynthesis of unsaturated fatty acids, and ether lipid metabolism. The genes and pathways primarily came from bacteria in the *Nocardiaceae*, *Micrococcaceae*, *Corynebacteriaceae*, and *Desulfobacteraceae* families^35^. However, this type of information is largely unknown for other carnivores, especially felids.

In domestic cats, the major odor compounds of the anal gland are short-chain free fatty acids including acetic acid, propanoic acid, 2-methylpropanoic acid, butanoic acid, 3-methylbutanoic acid, and pentanoic acid. These profiles vary among individuals but not among sexes^42^. Although the bacterial taxonomic composition of the anal glands of true domestic cats has not yet been examined, in the Bengal cat (*Felis catus* × *Prionailurus bengalensis*) three microbes isolated from the anal gland produce many of the same chemical volatiles found in the secretions^34^. These three microbes—*Bacteroides fragilis*, *Tessarococcus* spp., and *Finegoldia magna*—were also abundant in the accompanying 16S rRNA gene sequence data. The combined evidence from these two prior studies suggests that several bacterial species in the felid scent gland could be producing odorant molecules that are employed by their host during chemical communication.

In this study, we built on these findings and used culture-dependent techniques in combination with shotgun metagenomics and metabolomics (thermal desorption-gas chromatography-mass spectrometry, TD-GC–MS) to examine anal gland secretions in twenty-three cats (N = 23; Table 1) that were evaluated at a tertiary referral hospital. Some of these cats had been previously diagnosed with chronic enteropathy, renal or intranasal disease but were asymptomatic and clinically healthy at the time of sampling. More importantly, their anal glands were healthy and free of abscesses, impactions, or infections. In this study, we profiled the bacterial and volatile components of anal gland secretions, and determined whether differences are found among cats of distinct ages, body conditions, living environments, diets, or a medical diagnosis of periodontitis, since bacteria could be exchanged between mouth and anal gland via grooming. Furthermore, we reported the extent to which microbiome and VOC profiles were correlated with one another and identified microbiome metabolic pathways potentially involved in VOC synthesis. Lastly, we reconstructed metagenome-assembled genomes (MAGs) from shotgun sequence data which can be further annotated to advance our understanding of the diversity and functions of microbes involved in host chemical communication.

**Table 1:** Characteristics of the twenty-three domestic cats (N = 23) that participated in this study.

Characteristic	Subcategory	Number of cats (N = 23)
Age, in years		Range: 2–14 Median: 6.9
Body condition (1–9)	3–4	4 (17%)
	5–6	10 (43%)
	7	5 (22%)
	8–9	4 (18%)
Sex	Female	12 (52%)
	Male	11 (48%)
Spayed or neutered	Yes	23 (100%)
Breed	Domestic shorthair	19 (83%)
	Other	4 (17%)
Diet	Dry kibble only	13 (57%)
	Dry kibble and canned food	6 (26%)
	Other	4 (17%)
Environment	Indoor only	16 (70%)
	Indoor and outdoor	7 (30%)
Antibiotics (within prior 6 months)	Yes	1 (4%)
	No	22 (96%)
Periodontal disease diagnosis (moderate to severe)	Yes	9 (39%)
	No	14 (61%)
IBD diagnosis	Yes	3 (13%)
	No	20 (87%)

Samples were collected from the anal glands of cats that were presented to the UC Davis Veterinary Teaching Hospital for elective procedures that required sedation or general anesthesia. Health and lifestyle data on each individual cat is summarized above. The full list of samples and their metadata are provided in Table S1.

## Methods

### Sampling the anal gland and perianal region of domestic cats

Companion cats (N = 23) that presented to the Veterinary Medical Teaching Hospital at UC Davis between December 2021 and March 2022 for elective procedures were evaluated for enrollment in the study. The elective procedures included dental cleaning, abdominal ultrasounds, radiographs, or oral examinations that required sedation or general anesthesia. Sedation or general anesthesia was performed by anesthesiologists and clinicians using established protocols (e.g. Dexmedetomidine at 4 mcg/kg IV and Methadone at 0.2 mg/kg IV for sedation, or Maropitant at 1 mg/kg IV and Butorphanol at 0.2–0.4 mg/kg IM ). After obtaining informed written consent from cat owners, a board-certified internist and gastroenterologist (S.L.M) manually expressed the anal glands of cats while they were sedated or anesthetized by inserting a lubricated gloved index finger into the cat’s anus and digitally squeezing the anal gland between the index finger and thumb into a sterile 2″ × 2″ gauze sponge. Collected anal gland material was immediately transferred onto three sterile Puritan cotton swabs (one for microbiome analysis, one for microbial culturing, and one for metabolomics). In addition, a swab from the cat’s perianal region was collected for comparison to the microbiome found in the anal gland. Finally, an unused Puritan cotton swab (blank negative control) and a swab of the internist’s examination glove prior to the procedure were collected as well to assess background contamination.

The swabs were placed in 2 mL screw cap tubes (for microbiome analysis) or 20 mL borosilicate glass vials (for metabolomic analysis), and stored at − 80 °C until laboratory and chemical analysis. For culturing, swabs were also placed in 2 mL tubes but were never frozen or refrigerated since they were immediately taken to the lab for agar plating. After sample collection, the cat’s perianal region was cleaned with warm water and sprayed with a feline deodorizer. The cat was closely monitored and observed during recovery. Data on the animal’s signalment (age, breed, sex), health status (including any medical diagnoses), lifestyle (living environment), body weight, body condition, and diet were also collected (Table S1). With the exception of a single cat, individuals had not been treated with systemic antibiotics within 6-months of enrollment in the study. We kept this cat in our dataset as its microbiome was not compositionally anomalous in any obvious way compared to those from other surveyed cats.

The study was approved by the University of California, Davis, Institutional Animal Care and Use Committee (IACUC protocol # 22528). All methods were performed in accordance with the relevant guidelines and regulations, including the ARRIVE guidelines (Animal Research: Reporting of In Vivo Experiments).

### Bacterial culturing and isolation from the anal gland

Bacterial cultures of the anal gland were obtained from 11 of the 23 cats (Table S1); we did not culture microbes from the remaining 12 cats due to timing. Shortly after sample collection, bacterial swabs from the anal gland were vortexed with 1 mL of PBS and two serial 1:10 dilutions were performed. For each mixture, 150 µL was pipetted into lysogeny broth (LB), brain heart infusion (BHI), and blood agar (BA) plates. The plates were placed in BD GasPak EZ Anaerobic System boxes (BD Biosciences, NJ, USA) with packets of CO_2_ generators to maintain an anaerobic environment. After growing plates at 37 °C for 3–5 days, colonies with distinct morphologies, colors, and textures were picked from any or all of the three types of agar plates and plated until pure colonies were obtained. Single colonies were grown anaerobically in liquid broth for 3 days at 37 °C in preparation for DNA extractions.

### DNA extraction and Sanger sequencing of bacterial cultures

DNA was extracted from liquid bacterial broth cultures using the Wizard SV Genomic DNA Purification Kits (Promega, WI, USA), according to their protocol for Gram-negative bacteria. Briefly, 1.5 mL of vortexed broth was centrifuged and subsequently exposed to nuclei lysis, RNAse digestion, incubation at 37 °C, and protein precipitation on ice. The DNA was washed with isopropanol and ethanol, and the pellet was air-dried and then resuspended in nuclease-free water.

For each bacterial isolate, the 16S rRNA gene was amplified using the 27F (5′-AGAGTTTGATCMTGGCTCAG-3′) and 1391R (5′-GACGGGCGGTGTGTRCA-3′) bacterial-specific primers. The PCR conditions were as follows: an initial denaturation step at 95 °C for 3 min, followed by 30 cycles of 95 °C for 45 s, 50 °C for 60 s and 72 °C for 90 s. A final extension occurred at 72 °C for 10 min, and a final hold at 15 °C. PCR products were purified with the NucleoSpin Gel and PCR Clean-Up kit (Takara Bio, CA, USA) and quantified with Qubit HS dsDNA assay (Thermo Scientific, MA, USA).

A total of 111 bacterial isolates were submitted for Sanger sequencing of the 16S rRNA gene (27F primer) at the UC Davis College of Biological Sciences DNA Sequencing Facility (Davis, CA, USA). Sanger chromatograms were uploaded to myRDP^43^ for quality-trimming and base-calling. The trimmed sequences were searched against the bacterial NCBI RefSeq genomes database^44^ using blastn for taxonomic identification, setting default parameters. The top hit with the highest e-value and percent identity was selected as that organism’s taxon label (Table S18). Six of the 111 isolates did not meet sequence quality thresholds and could not be classified taxonomically.

### DNA extraction and metagenomic sequencing of bacterial swabs

Genomic DNA was extracted directly from swabs of the anal gland (N = 23), perianal region (N = 6), and controls (a sterile swab and swab of glove; N = 2) using the QIAGEN DNeasy Powersoil Pro Kits (Qiagen, MD, USA) (Table S1). Swabs were incubated with the QIAGEN CD1 lysis buffer at 65 °C for 10 min and then underwent bead-beating for 1.5 min before resuming the manufacturer’s protocol for this kit. Genomic DNA from 31 samples was treated with RNAse A and sequenced on an Illumina NextSeq 500 at the UC Davis Genomics Core to generate PE × 150 bp reads.

### Sequence processing of metagenomic data

Sequenced samples had an average of 5,527,421 (± 997,985) metagenomic paired-end reads which were quality-filtered and trimmed using Trimmomatic v.0.38 setting default parameters^45^. On average, samples retained 95% (± 1.07%) of their sequences after quality-filtering (Table S2). Reads were filtered of host DNA by aligning them to two *Felis catus* reference genomes (GenBank accessions GCA_000181335.5 and GCA_013340865.1) using Bowtie2 (v.2.4.2)^46^. Kraken 2 (v.2.1.2)^47^ assigned taxonomic classification to the host-filtered quality-trimmed reads using default parameters and its standard database that included bacterial, Archaeal, Eukaryotic, and viral sequences. Bracken (Bayesian Reestimation of Abundance with Kraken)^48^ estimated the taxon abundances at the Family, Genus, and species level. (Table S2). When Kraken was not able to assign a species label to a sequence, it used the genus or family label followed by spp. (e.g. *Bacteroides* spp.).

The resulting forward and reverse metagenomic reads were interleaved using a python script from the Ray assembler v.2.3.1^49^ and concatenated into a single file in preparation for metagenome assembly. Sequences were assembled into contigs with (meta)SPAdes v.3.14.1^50^ and the quality of assembly was evaluated with QUAST v.5.0.0^51^ (Table S3). After removing contigs shorter than 300 bp using BBMap v.38.87, contigs were uploaded into Anvi’o v.7.1^52^ for gene prediction and functional annotation. Gene prediction was accomplished with Prodigal v.2.6.3^53^, and functional annotation was done using the Clusters of orthologous groups (COGs)^54^, and the Kyoto Encyclopedia of Genes and Genomes (KEGG) databases^55^. To determine the abundances of genes in each sample, host-filtered reads were mapped to predicted genes using Salmon (v.1.8.0)^56^. Gene relative abundances were in units of Transcripts Per Million (TPM), which normalizes for both gene length and sample sequencing depth. On average, 70.35% of host-filtered reads mapped to putative ORFs (range: 46–79%) (Table S2). Lesser percentages were assigned an actual KEGG or COG annotation (66% of mapped reads for COG, ~ 46% of mapped reads for KEGG).

Contigs with a minimum length of 600 bp were binned into metagenome-assembled genomes (MAGs) using MetaBat2 v.2.15^57^. A total of 85 high-quality MAGs were recovered with completeness scores > 80% and contamination scores < 5% as assessed by CheckM (v.1.1.3)^58^ (Table S4). The Genome Taxonomy Database Toolkit (GTDB-Tk) (v.1.5.0)^59^ was used to assign taxonomic identity to MAGs using database release 202^60^. The abundance of each MAG in a sample was estimated using CoverM (v.0.6.1) (https://github.com/wwood/CoverM) by mapping interleaved host-filtered reads to each MAG (Table S5). On average, 60.37% of reads in each sample were able to be mapped to MAGs (range: 32–88%) (Table S2). A phylogeny of these MAGs was constructed with RAxML (v.8.2.11)^61^ using the multiple-sequence alignments generated by GTDB-Tk. We had no outgroup and instead rooted our tree to the only member of the phylum *Synergistota* (*Fretibacteriu*m).

### Extraction and analysis of anal gland metabolites

Volatile compounds were extracted from anal swab samples using two techniques. First, solid phase microextraction (SPME) fibers (50/30 µm DVB/CAR/PDMS coating) were exposed to swabs in vials to extract VOCs from the headspace. Then, VOCs were extracted from swabs using liquid phase extraction with methanol, followed by a derivatization process before being directly analyzed.

For headspace technique, a 1 µL aliquot of 10 mL/L decane-d22 was added to the 20 mL borosilicate glass vials containing the swabs as an internal standard. Two previously conditioned SPME fibers were exposed to anal swabs for 24 h at room temperature, then capped and placed in a − 20 °C freezer until spectrometric analysis. For the liquid phase extraction, these same swabs were placed into 20 mL of methanol for 24 h at room temperature to extract VOCs into solution. A 2 mL aliquot of each extract was transferred into a new vial and completely dried under nitrogen. Dried extracts were subsequently derivatized by adding 50 µL MTBSFTA and 50 µL acetonitrile. Reconstituted samples were left to react for 1 h at 60 °C and stored at − 20 °C until spectrometric analysis.

VOCs from SPME fibers and derivatized extracts were analyzed with gas chromatography-mass spectrometry. For SPME fibers, the fiber was inserted into the inlet of an Agilent 6890N gas chromatograph (Agilent Technologies Inc.) set to 260 °C. VOCs were desorbed from the fiber for 5 min in splitless mode while the GC oven was held at 40 °C. For liquid extracts, 1 µL of each sample was injected into the GC inlet held at 260 °C. For both samples, the oven was ramped to 120 °C at 5 °C/min, and then ramped to 280 °C at 15 °C/min, holding for 10 min. VOCs were separated on a DB-5 ms column (30 m × 250 μm × 0.25 μm, Agilent Technologies Inc.) with a 1 mL/min constant flow of helium. Compounds were eluted through a 300 °C transfer line into an Agilent 5795C mass spectrometer, which scanned 50 to 500 m/z with its source set to 230 °C and quadrupole to 150 °C.

Samples and blanks/controls were injected in a random order to produce reliable data. A standard Grob mixture was injected in triplicate to monitor instrument performance, and a standard mix of C_8_-C_30_ alkanes was analyzed to calculate the Kovats Retention Indices of each VOC. Raw data were first checked for qualitative reasons using Agilent’s Mass Hunter Qualitative Analysis B.06.00 software. GC–MS data were then deconvoluted and aligned using the recursive feature extraction on Profinder (Version B.08.00, Agilent Technologies Inc.) and Mass Profiler Professional (MPP, V13.0). An initial table was then obtained containing all peak intensities (rows) from each sample (column). Peaks from contaminants like siloxanes (base peaks 207, 221 and 281 m/z) were removed. Features that appeared in blanks with a signal more than five times the signal from samples (peak sample/blank ratio) were removed. These blanks were composed of system blanks (instrumental blank without injection), Twister^®^ blanks (injection of clean twisters), blank vials and blank cotton swabs; the latter two were treated as if they were biological samples and underwent SPME extraction and liquid extraction as described above.

Compounds were tentatively identified by matching the mass spectra with structures available in the National Institute of Standards and Technology (NIST) 2020 Library and by matching calculated retention times with those reported in the literature.

### Statistical analysis of microbiome and metabolome data

Unless otherwise stated, all sequence data was analyzed and visualized using the R statistical software program (v4.3.0)^62,63^. Prior to any statistical analysis, we used R decontam (v.1.20.0)^64^ to identify and remove contaminant bacterial taxa from the Kraken2 dataset based on their prevalence in control samples compared to samples from the anal gland and perianal region. A total of 58 bacterial species (Table S6) were deemed contaminants by decontam (had scores below the specified threshold of 0.5) and were removed. Kraken read counts taxonomically assigned to “*Homo*”, viruses, and Archaea were also removed.

The composition of bacterial communities from the felid anal gland (or the perianal region) was visualized using data generated by Kraken2/Bracken (Tables S7, S8). For this, bar plots showing the relative abundances of bacterial families and genera were constructed using ggplot2 (v.3.4.2)^65^. Next, we examined whether five host factors of interest: age (in yrs), obesity (obese vs. not obese), living environment (indoor vs. indoor and outdoor), diet (dry food only vs. other diets), and a medical condition (moderate to severe periodontal disease vs. no disease) could account for any of the variance in anal gland microbiome beta-diversity. For analyses, cats with body condition scores (BCS) 8–9 were considered “obese” and all other cats were classified as “not obese”^66^. This classification was selected because of its clinical relevance and the multitude of obesity-associated comorbidities.

Genus-level abundance data generated by Kraken2/Bracken was converted to presence/absence (for Jaccard dissimilarity), proportions (for Bray–Curtis dissimilarity), or applied a Center-Log-Ratio (clr) transformation (for Aitchinson dissimilarity). Permutational multivariate analyses of variance (PERMANOVAs) tested whether microbiome beta-diversity varied with the five host factors. Tests evaluated all factors simultaneously, in a way where the order of terms did not influence statistical output (e.g. they were marginal PERMANOVA tests). PERMANOVAs employed 999 permutations, set alpha to 0.05, and were conducted with the vegan package (v.2.6-4)^67^.

We investigated whether microbiome profiles from the anal gland were significantly correlated with metabolome profiles (from solid-phase and liquid-derivatization extractions). For this, the matrix of metabolite absolute abundances (Table S9 for solid extraction, Table S10 for liquid extraction) were normalized by converting to relative abundances (e.g. proportions), log_10_-transformed to minimize the influence of heteroscedasticity, and scaled with pareto scaling, which is advised for metabolite data^68^. Metabolite Jaccard and Euclidean distances were estimated using the phyloseq package (v.1.44.0). Mantel tests correlated microbiome matrices (Jaccard, Bray–Curtis and Aitchison) to metabolite Jaccard or Euclidean distances using 999 permutations. Because a statistically significant relationship existed between the two datasets, we plotted this relationship; specifically, we extracted the first two principal coordinates from metabolite and microbiome dissimilarity matrices using the cmdscale function from the stats package (v.4.3.0)^62,63^.

We also tested whether the relative abundances of bacterial species were significantly associated with the relative abundances of specific metabolites using Spearman correlations (R stats package). One correlation test consisted of 1 bacterial genus and 1 metabolite, and were repeated until bacterial genera were compared against all metabolites. P-values were adjusted for multiple comparisons using the False Discovery Rate (FDR). Only bacterial species found in at least 90% of anal gland samples and at a mean relative abundance > 0.2% were considered. For metabolites, all 37 putatively identified metabolites were considered, along with 187 unidentified metabolites that had normalized and scaled mean relative abundances > 0.

Analyses on COG and KEGG ortholog and pathway abundances were also conducted (Tables S11–S14). Gene abundances which were estimated in TPM were normalized using total sum scaling. PERMANOVAs examined whether microbiome functions were significantly predicted by host factors, using the methods described above. Mantel tests evaluated whether microbiome functional profiles were correlated with metabolite abundance data as described earlier for the microbiome-metabolite analyses.

All the methods described previously in this section were also used to analyze metagenome-assembled genomes (MAG) abundances. PERMANOVAs tested whether MAG relative abundances (Table S5) or their presence/absence were significantly associated with host age, obesity, living environment, diet, and a medical diagnosis of periodontitis. Mantel tests evaluated whether MAG profiles were associated with metabolite profiles. Spearman correlations were used to correlate the abundances of all MAGs classified to species level (47 MAGs) to the abundances of 37 putatively identified metabolites and 193 unidentified metabolites. P-values were adjusted for multiple comparisons using FDR.

Lastly, marginal PERMANOVA tests were also employed to test whether the taxonomic and functional compositions of microbiomes in the anal gland were distinct from those in the perianal region.

## Results

### Characteristics of feline study participants

The anal gland microbiomes and metabolomes of 23 cats were surveyed for this study; six of these cats also had corresponding metagenome sequences from the perianal region. Cat participants were predominantly indoor domestic shorthairs, all spayed or neutered, ranging in age from 2 to 14 years old (Tables 1, S1). There were equal proportions of males and females. Forty percent of cats were overweight or obese (BCS 7–9) (Tables 1, S1). Over half of the surveyed cats (57%) were only fed dry kibble, and the remaining were fed canned food and dry kibble, or had other diets. Thirty-nine percent of study participants had been previously diagnosed with moderate to severe periodontal disease and 13% were diagnosed with chronic enteropathy (IBD or intestinal lymphoma), though at the time of sampling, all cats were clinically healthy on physical examination. None of the cats had diarrhea when they were evaluated and none had evidence of anal gland disease.

### Which bacterial taxa inhabit the anal gland in cats?

One key goal of this study was to survey the taxonomic, functional, and chemical composition of the anal gland microbiome in twenty-three domestic cats (Table 1), and in doing so, shed light on the bacteria or bacterial gene pathways potentially involved in the production of volatile organic compounds (VOCs) likely being used by their host for chemical communication. The first step was to provide a snapshot of the microbiome composition in the anal gland.

**Figure 1:**
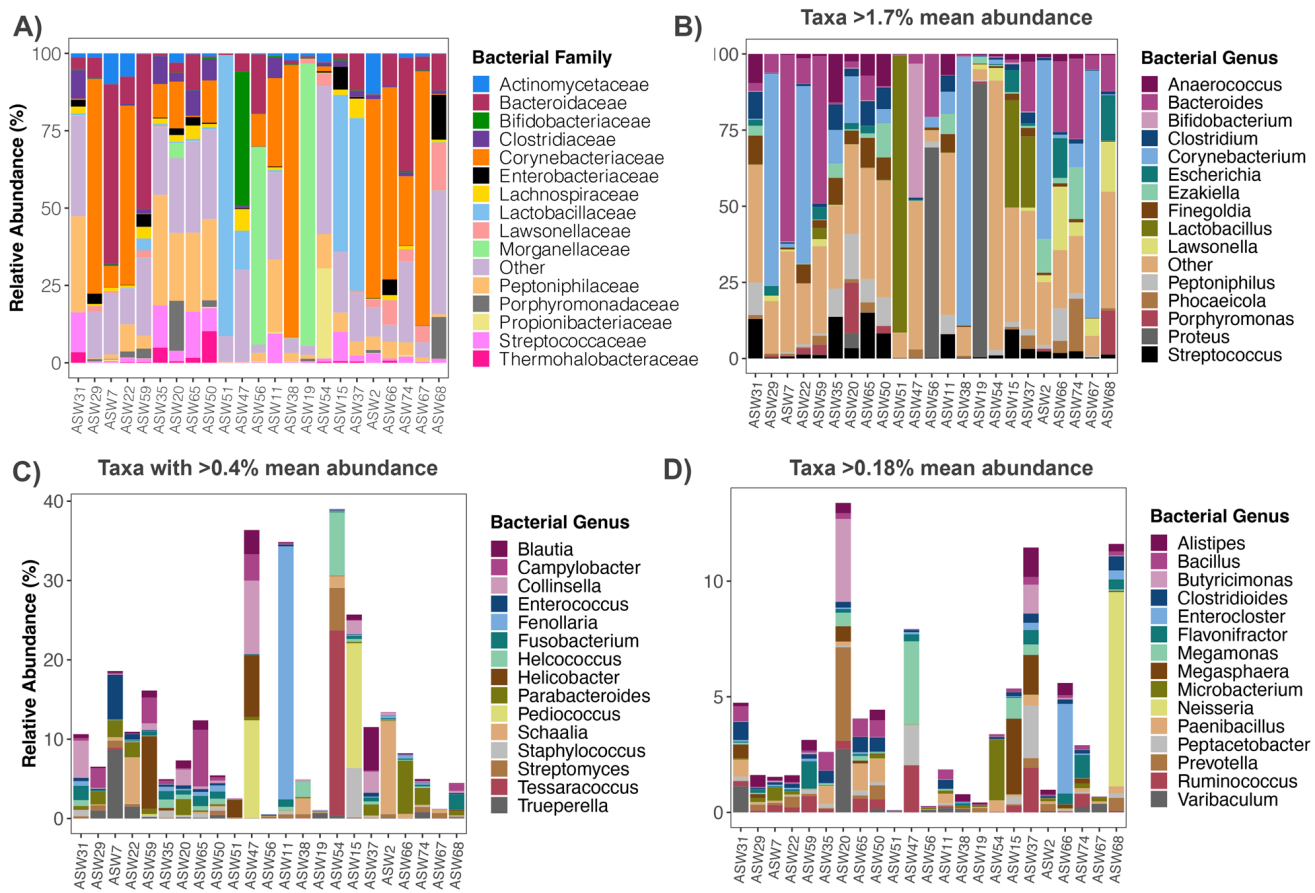
Bacterial taxonomic composition of the anal gland microbiome in domestic cats. Relative abundances of bacterial (**A**) families and (**B**–**D**) genera in anal gland metagenomes, as estimated using Kraken2/Bracken. Families with a mean relative abundance > 1% across samples are displayed. Genera with a mean relative abundance > 1.7% are shown in (**B**), > 0.4% but less than 1.7% are shown in (**C**), and > 0.18% but less than 0.4% are shown in (**D**).

The majority of metagenomic sequences recovered were classified as Bacteria (94% mean relative abundance) by the software Kraken2/Bracken, with the remainder of sequences classified as Eukaryotes (5% mean relative abundance), and Archaea and Viruses (< 1% combined mean relative abundance); though this could partly be due to biases in the reference database. The most represented bacterial families in feline anal glands included *Corynebacteriaceae* (23% mean relative abundance), *Bacteroidaceae* (11%), *Peptoniphilaceae* (10%), and *Lactobacillaceae* (9%) (Fig. 1A, Table S7). The single most frequently detected bacterial genus in sequencing reads was *Corynebacterium*, which on average constituted 18% of the microbiome in the anal gland. *Bacteroides* (11% mean relative abundance), *Proteus* (7%), *Lactobacillus* (7%), *Streptococcus* (4%), and *Peptoniphilus* (4%) had moderately high relative abundances (Fig. 1B, Table S8).

The microbiome compositions of the anal glands of these cats were highly variable among individuals. The anal gland microbiomes of three cats for example were almost exclusively composed of *Corynebacterium* (> 70% relative abundance), while the anal gland microbiomes of other cats were dominated by *Proteus* (> 70% relative abundance) or *Bacteroides* (25–60% relative abundance) (Fig. 1B). The anal gland of one cat almost exclusively contained *Lactobacillus johnsonii* (88% relative abundance), and in other samples, this same bacterial taxon was rare (< 3% relative abundance). This type of individual variation was also evident when examining the abundances of bacterial genera. One individual, a domestic long-haired cat, had an anal gland where *Porphyromonas gingivalis* constituted 13% of the community (Fig. 1C, sample ID ASW68). *Enterococcus* (mainly comprised by *E. avium*) constituted 4% of the anal gland microbiome in an 8-year old cat with low-grade intestinal lymphoma (sample ID ASW7), and this bacterial group was virtually absent from the anal glands of other cats (Fig. 1D). The anal glands of the smallest cat in the dataset (sample ID ASW20) which ate only canned food, housed the highest relative abundances of the butyric-acid producing bacteria *Butyricimonas virosa* (3%) and of *Prevotella copri* (4%) (Fig. 1D). However, due to the study’s small sample size, associations between host lifestyle factors and bacterial abundances should be interpreted with caution.

**Figure 2:**
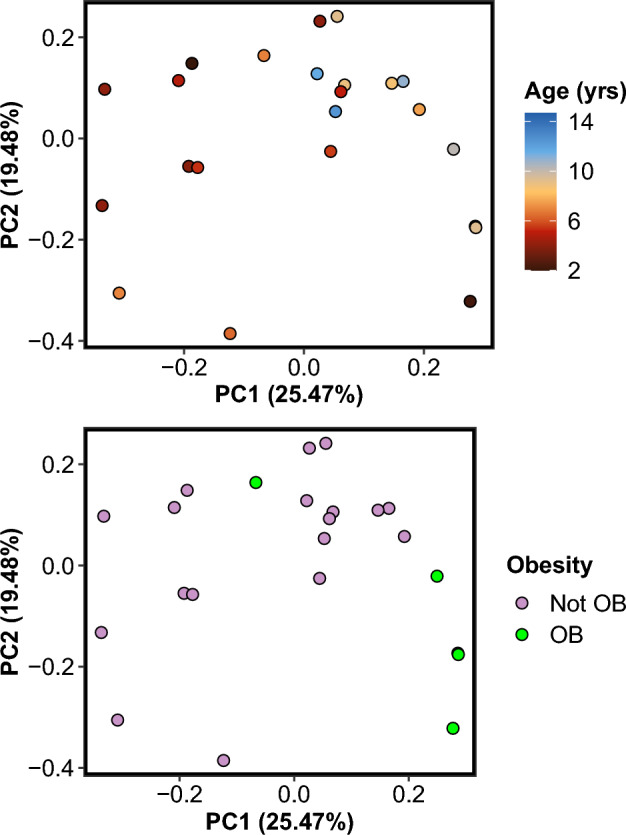
Anal gland microbiomes are significantly correlated with host age and obesity category. PCoA plots were constructed from Jaccard dissimilarity matrices based on bacterial genus-level relative abundances estimated from shotgun Illumina sequence data. Each point represents a sample and is color-coded by cat age in years (top) or obesity category (bottom). *OB* obese.

Next, we asked whether any of the observed variation in bacterial community composition could be explained by host characteristics, including age, obesity, living environment, diet , and a medical diagnosis of periodontitis. Anal gland microbiome beta-diversity was significantly associated with host age and obesity (Fig. 2); these predictors accounted for 12% and 8% of the variance in microbiome, respectively (PERMANOVA, Table 2). Specifically, the anal gland microbiome compositions of older cats were generally distinct from those of younger cats. The anal gland microbiomes of obese cats differed from those of non-obese cats, though our sample size was limited to 4 obese cats and future studies should evaluate this question with a larger dataset (Fig. 2, Table 2).

**Table 2:** Do host characteristics predict bacterial, metabolite, and functional pathway abundances in the anal gland?

Data type	Distance type	Age (yrs)	Obesity (obese vs. not)	Living environment (indoor vs. indoor-outdoor)	Diet (dry food only vs. other)	Medical diagnosis (has periodontitis. vs not)
Microbiome	Jaccard	**0.092 (p = 0.013)**	**0.087 (p = 0.034)**	0.054 (p = 0.18)	0.029 (p = 0.77)	0.024 (p = 0.9)
	Bray–Curtis	**0.12 (p = 0.003)**	0.029 (p = 0.74)	0.043 (p = 0.37)	0.042 (p = 0.43)	0.034 (p = 0.57)
	Aitchison	0.068 (p = 0.09)	0.041 (p = 0.47)	0.055 (p = 0.20)	0.054 (p = 0.22)	0.046 (p = 0.35)
Metabolome (solid-phase)	Jaccard	0.016 (p = 0.95)	0.017 (p = 0.91)	0.029 (p = 0.67)	0.057 (p = 0.27)	0.033 (p = 0.61)
	Euclidean	0.025 (p = 0.91)	0.028 (p = 0.86)	0.035 (p = 0.61)	0.06 (p = 0.2)	0.039 (p = 0.5)
Metabolome (liquid-derivatization)	Jaccard	0.051 (p = 0.33)	0.045 (p = 0.39)	0.057 (p = 0.26)	0.019 (p = 0.88)	0.035 (p = 0.57)
	Euclidean	0.044 (p = 0.41)	0.039 (p = 0.50)	0.061 (p = 0.17)	0.024 (p = 0.86)	0.037 (p = 0.54)
COGs functions	Jaccard	0.049 (p = 0.24)	0.049 (p = 0.29)	**0.12 (p = 0.025)**	0.026 (p = 0.7)	0.021 (p = 0.79)
	Bray–Curtis	**0.13 (p = 0.005)**	0.045 (p = 0.32)	0.04 (p = 0.4)	0.038 (p = 0.44)	0.029 (p = 0.63)
	Aitchison	**0.085 (p = 0.01)**	0.045 (p = 0.32)	0.05 (p = 0.23)	0.041 (p = 0.43)	0.037 (p = 0.56)
KEGG functions	Jaccard	0.045 (p = 0.31)	0.045 (p = 0.33)	**0.11 (p = 0.039)**	0.022 (p = 0.8)	0.019 (p = 0.86)
	Bray–Curtis	**0.13 (p = 0.008)**	0.037 (p = 0.43)	0.045 (p = 0.29)	0.036 (p = 0.44)	0.028 (p = 0.64)
	Aitchison	**0.092 (p = 0.01)**	0.041 (p = 0.46)	0.05 (p = 0.21)	0.042 (p = 0.41)	0.035 (p = 0.61)
Metagenome-assembled genomes (MAGs)	Jaccard	0.051 (p = 0.23)	0.040 (p = 0.58)	**0.068 (p = 0.04)**	0.045 (p = 0.41)	0.045 (p = 0.4)
	Bray–Curtis	**0.10 (p = 0.004)**	0.038 (p = 0.49)	0.058 (p = 0.11)	0.041 (p = 0.47)	0.36 (p = 0.61)
	Aitchison	**0.085 (p = 0.014)**	0.027 (p = 0.91)	0.045 (p = 0.43)	0.036 (p = 0.7)	0.034 (p = 0.75)

R^2^ and p-values for PERMANOVAs that correlated five host predictors (all in a single model) with Genus-level bacterial relative abundances, metabolite relative abundances, metagenomic gene pathway abundances, or MAG abundances. Significant p-values (a = 0.05) are bolded.

### Do metabolite profiles correlate with microbiome profiles?

A total of 428 metabolites were detected using solid phase microextraction (Table S9), of these 37 (8.6%) were putatively identified. Among the identified metabolites were fatty acids (nonanoic acid, hexadecanoic acid), esters (2-methylbutanoic acid, benzoic acid ethyl ester, pentanoic acid 4-methyl-), aldehydes (benzaldehyde, propanal), ketones (acetophenone, cyclohexanone), and hydrocarbons (ethylbenzene). The putatively identified compounds with the largest average relative abundances across samples were the aromatic hydrocarbons styrene and ethylbenzene, and the aldehyde benzaldehyde.

For the liquid phase extractions, a total of 145 metabolites were detected, of which 51 (35.1%) were tentatively identified (Table S10). The derivatized samples contained cholesterol-related compounds, alcohols, and esters. The low number of putatively identified volatile organic compounds (VOCs) reflects a limitation of the libraries used to identify compounds, in that they are not as exhaustive with regards to bacterial or fungal-associated VOCs.

**Figure 3:**
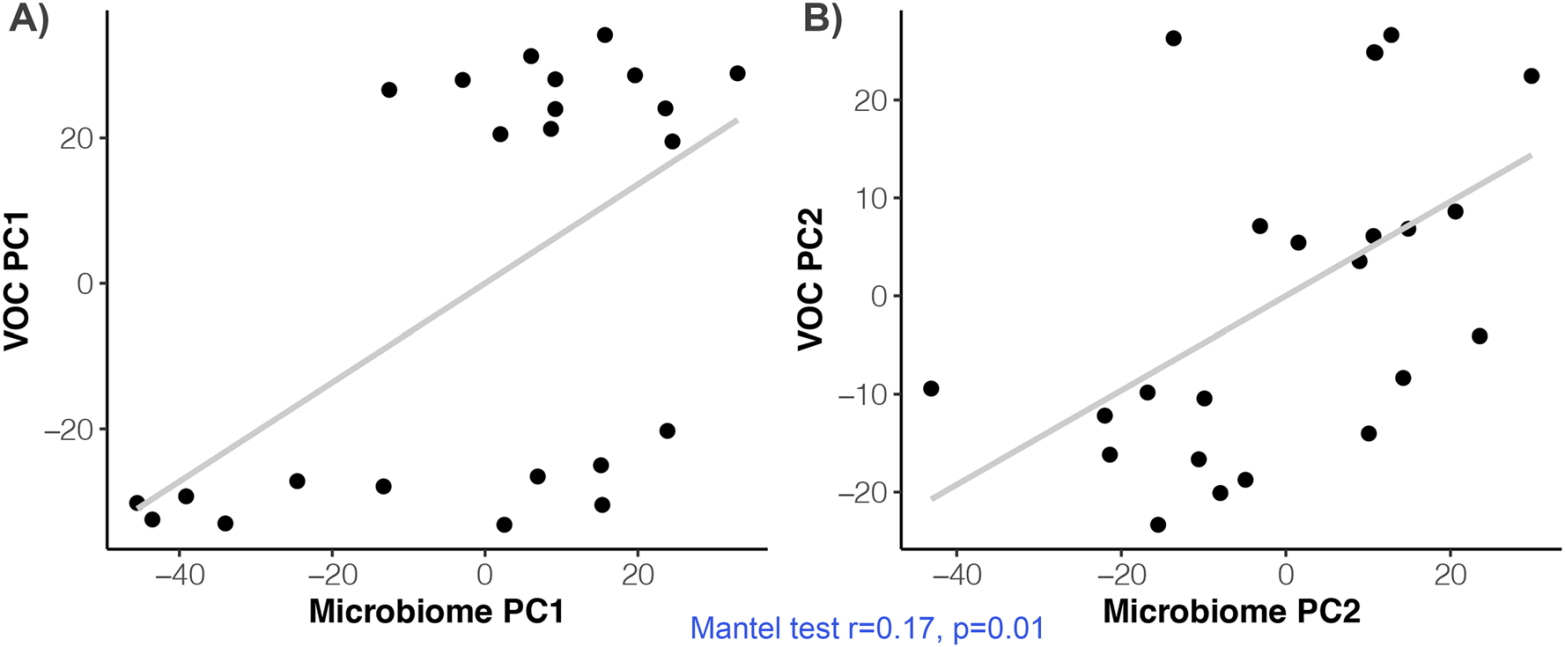
Microbiome profiles covary with metabolite profiles in the feline anal gland. Microbiome taxonomic profiles were estimated from shotgun Illumina sequence data; metabolite data were obtained using GC–MS with solid-phase microextraction. According to mantel tests, microbiome dissimilarity (Aitchison distance) was significantly correlated with metabolite dissimilarity (Euclidean distance).

We then tested whether a relationship existed between the bacteria residing in the anal gland and the metabolites found in the anal gland. Microbiome and metabolome profiles detected during solid-phase microextraction were modestly positively correlated when using Aitchison distance for microbiome data (Mantel test r = 0.17, p = 0.01) but not when using Bray–Curtis (Mantel test r = 0.05, p = 0.23) or Jaccard distances (Mantel test r = 0.05, p = 0.26) (Fig. 3). Microbiome profiles were not significantly associated with metabolome profiles estimated after liquid derivatization (Mantel test Jaccard r = 0.09, p = 0.21; Bray–Curtis r = − 0.04, p = 0.71; Aitchison r = − 0.003, p = 0.51). Additionally, metabolome profiles did not vary with any of the host characteristics examined (Table 2).

Next, we ascertained whether the relative abundances of specific bacterial species were significantly correlated with the relative abundances of specific metabolites detected during solid-phase extractions since no relationship was found between microbiome profiles and metabolite profiles after liquid derivatization. Two bacterial species had relative abundances that were correlated with the most metabolites, and many of those correlations were negative correlations with unidentified metabolites (Spearman correlations, Table S15). These bacteria were *Clostridioides perfringens* (rho = − 0.5, p < 0.05) and *Streptococcus equi* (*r* = − 0.6, p < 0.05) (Table S15). The relative abundances of five bacterial species were positively correlated with nonanoic acid—a fatty acid, and these taxa were two *Clostridium* species (both *r* = 0.6, p < 0.05), *Fenollaria sporofastidiosus* (r = 0.6, p < 0.05), and two *Streptococcus* species (*r* = 0.7, *r* = 0.6, p < 0.05).

Eight bacterial species had relative abundances that were positively correlated with the relative abundances of a branched-chain fatty acid ester (2-methylbutanoic acid), including two *Clostridium* species (Spearman correlations, *r* = 0.5, *r* = 0.6, p < 0.05), three *Streptococcus* species (all *r* = 0.6, p < 0.05), two *Ezakiella* species (both *r* = 0.7, p < 0.05), and *Fenollaria sporofastidiosus* (*r* = 0.7, p < 0.05) (Table S15). *Clostridium septicum*, and *Fenollaria sporofastidiosus* were negatively associated with the relative abundances of epicholestanol and cholesterol (all *r* = − 0.6, p < 0.05).

### Are microbiome putative functions related to VOC synthesis?

To inspect the predicted metabolic functional repertoire of these microbiomes and identify putative functions potentially involved in VOC synthesis, we annotated genes predicted from metagenome contigs using the Cluster of Orthologous Genes (COGs) and the Kyoto Encyclopedia of Genes and Genomes (KEGG) databases. For both datasets, we obtained annotations of specific genes (COGs functions; KEGG orthologs) and of broader metabolic pathways (COGs categories; KEGG modules) (Tables S11, S13). Unfortunately, analysis of the most abundant COGs functions or KEGG orthologs (Tables S12, S14) across the dataset are not informative, as they mainly code for conserved proteins involved in bacterial growth and replication or are putative transposases of undetermined function. Instead we purely describe the genes that are related to fatty-acid, aldehyde, ketone, or alcohol metabolism, many of which were found at low relative abundances in anal gland samples.

We found genes predicted to code for alcohol dehydrogenases, which oxidize alcohols into aldehydes and ketones (or the reverse reaction), and aldehyde dehydrogenases, which oxidize aldehydes into carboxylic acids like acetic, propionic or valeric acid (Tables S12, S14). Genes encoding butanol dehydrogenases were also detected, which catalyze the conversion of butyraldehyde to butanol. A few putative genes also coded for alcohol-forming fatty acyl reductases which catalyze the reduction of thioesters to alcohols and are key enzymes involved in the microbial production of fatty alcohols. Microbes in the anal gland also contained genes predicted to encode acetyl CoA acetyltransferases, which are one of several proteins involved in the oxidation of fatty-acids into ketone bodies (Table S12).

**Figure 4:**
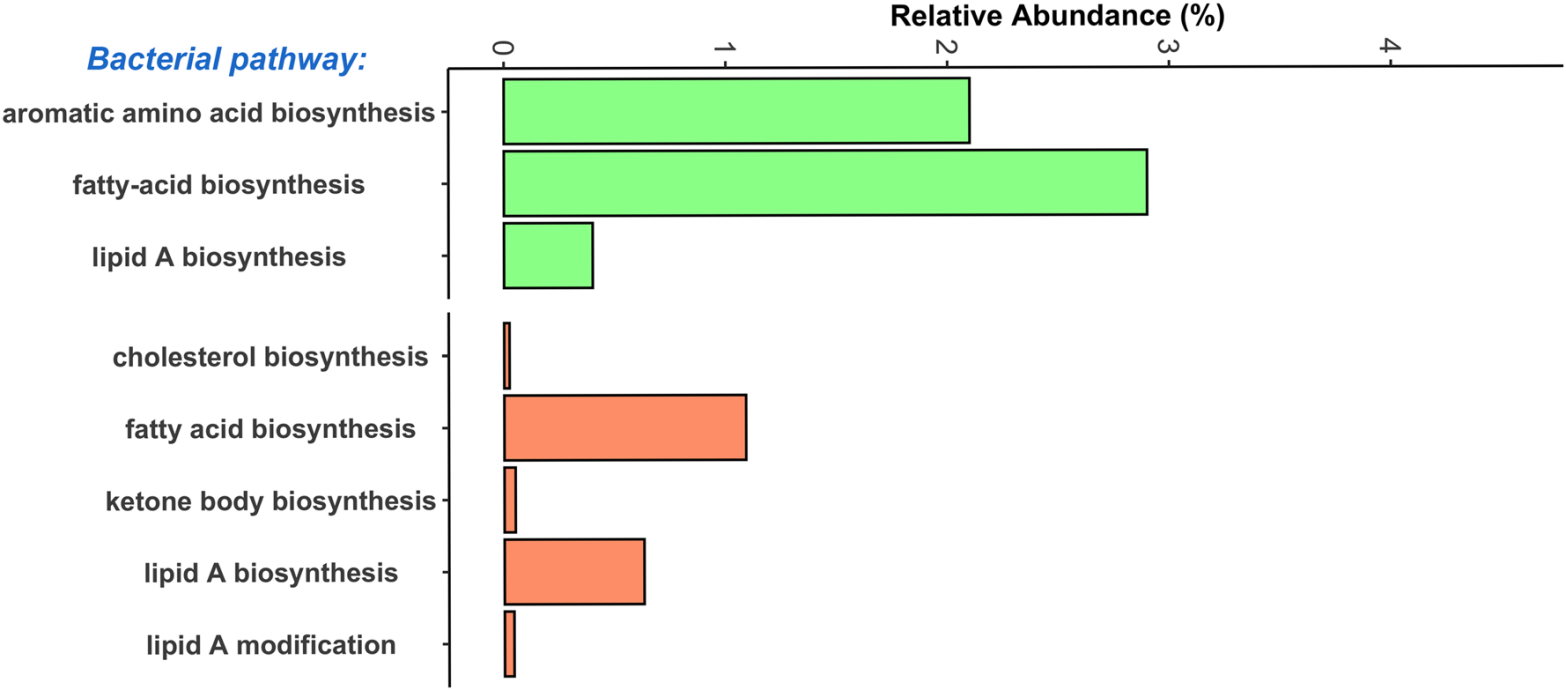
Relative abundance of microbial gene pathways potentially involved in VOC synthesis. Metagenome assemblies were functionally annotated with Anvi’o against the Cluster of Orthologous Genes (COGs) or Kyoto Encyclopedia of Genes and Genomes (KEGG). The abundance of shotgun Illumina sequence data mapping to each pathway was calculated in TPM and converted to relative abundances.

At a broad level, there were also bacterial pathways that encompassed functions relevant to the synthesis of volatile compounds. Out of 69 total COGs pathways in the dataset, three were relevant: “fatty-acid biosynthesis”, “lipid A biosynthesis”, and “aromatic amino acid biosynthesis” (Fig. 4). Fatty acid biosynthesis was the fifth most abundant pathway and aromatic synthesis was the 13th most abundant (Table S11). The KEGG modules with most direct relevance to VOC metabolism were ketone body biosynthesis, cholesterol biosynthesis, fatty acid biosynthesis, and lipid A biosynthesis and modification. These pathways were not as abundant as the COGs pathways (Fig. 4, Table S13).

Next, we examined whether microbiome functional profiles as a whole were significantly associated with host factors or metabolite abundances. We found that anal gland microbiome functional repertoires were significantly correlated with host age and living environment (PERMANOVAs, Table 2). Host age accounted for 9–13% of the variance in functional microbiomes (p < 0.01) while host environment explained 11% of the variance (p < 0.05, Table 2). These distinctions were not apparent when examining the abundances of broad functional pathways (Table S16).

Lastly, COGs functional profiles (but not KEGG profiles) were moderately correlated with metabolite profiles acquired during solid-phase extraction (Mantel test r = 0.11, p < 0.05, Table S17). No significant or meaningful relationships were observed between the relative abundances of specific functional pathways and specific metabolites (Spearman correlations; results not shown).

### Reconstruction of metagenome-assembled genomes from bacteria of interest

**Figure 5:**
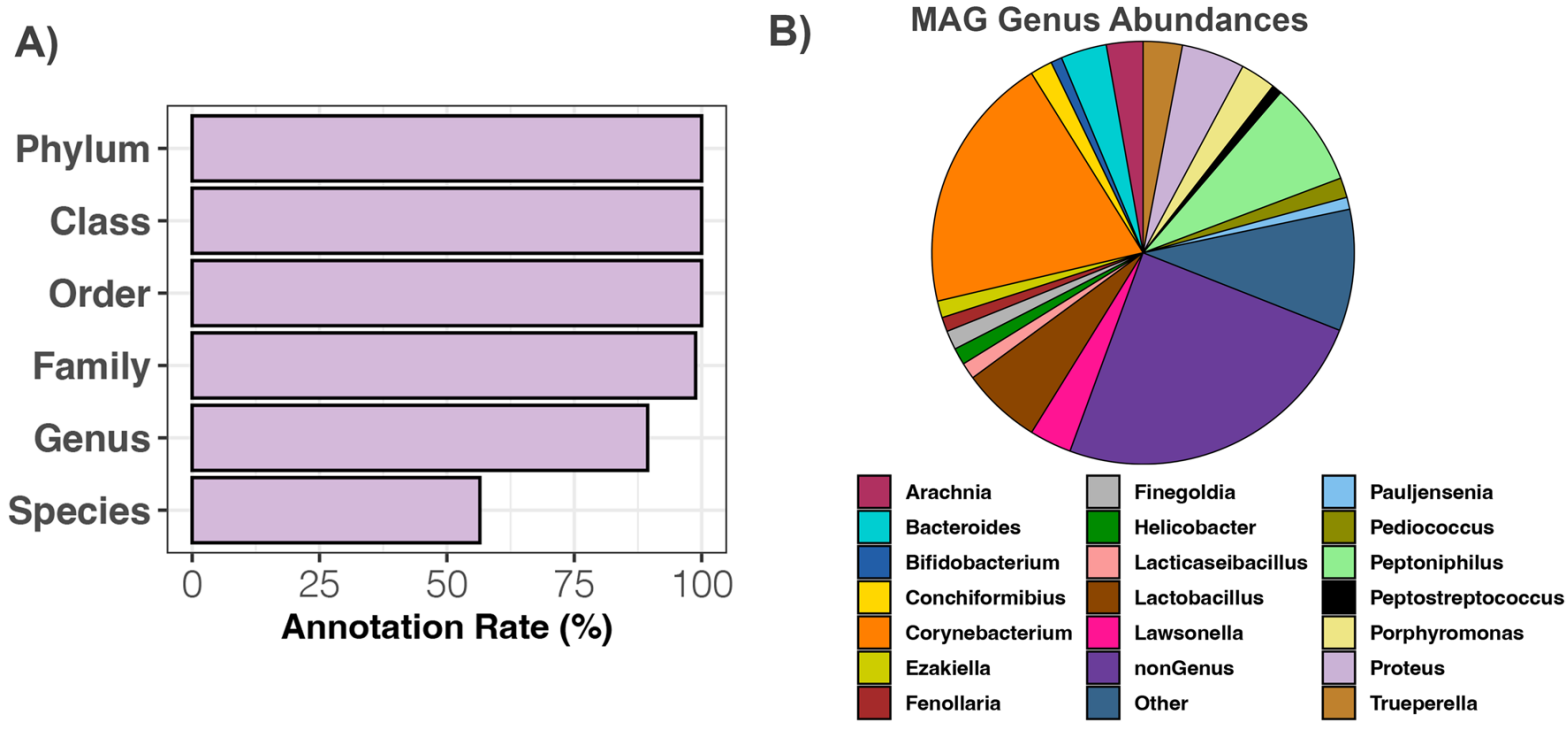
High-quality metagenome-assembled genomes (MAGs) reconstructed from the anal gland microbiome of domestic cats. (**A**) Annotation rate for the 85-quality MAGs (> 80% completeness and < 5% contamination) calculated by dividing the number of MAGs classified at that taxonomic level by the total number of MAGs. (**B**) Average relative abundances of MAG bacterial genera. ‘nonGenus’ represents the average summed relative abundances of MAGs that did not receive a Genus classification.

A total of 85 quality metagenome-assembled genomes (MAGs) were recovered from Illumina shotgun sequence data of the anal gland. These were on average 93.65% complete, and < 1% contaminated (Fig. S1, Table S4). Thirty-five bacterial families, fifty-three genera, and forty-seven species were represented. Close to 90% of MAGs were classified to genus level and over half (54%) were classified to species level (Fig. 5A).

Of the portion of Illumina shotgun metagenomic reads that could be mapped to MAGs, 19.8% of those reads were from MAGs classified to the genus *Corynebacterium* (Fig. 5B, Table S5). This number is highly comparable to that obtained from analyzing shotgun microbiome data (e.g. Kraken genus-level abundance data), where the abundance of *Corynebacterium* was 18%. Other bacterial taxa with high relative abundances in both MAGs and metagenomes included *Peptoniphilus* (7.9% MAG mean relative abundance vs. 4% Kraken mean relative abundance), *Lactobacillus* (6.08% MAG abundance vs. 7% Kraken abundance), and *Proteus* (4.83% MAG abundance vs. 7% Kraken abundance). *Bacteroides* was much more represented in the metagenome dataset (11% mean relative abundance) than as MAGs (3.5% mean relative abundance) (Fig. 5B).

The six MAGs with the highest mean relative abundances across anal gland metagenomes were: MAG #144 *Peptoniphilaceae* (22.5%), MAG #19 *Corynebacterium frankenforstense* (8.9%), MAG #124 *Peptoniphilus* (6.5%), MAG #138 *Lactobacillus johnsonii* (6.08%), MAG #57 *Corynebacterium pyruviciproducens* (5.39%), and MAG #5 *Proteus mirabilis* (4.8%) (Table S5). All other MAGs had mean relative abundances of < 4%. The *Peptoniphilaceae* MAG was most closely related to genomes belonging to several *Anaerococcus* species in the GTDB r202 database (Fig. S2). The closest relative to MAG #19 was a *C. frankenforstense* isolated from raw cow’s milk. The *Peptoniphilus* MAG #124 is closely related to a *P. lacydonensis* isolated from the human sinus (Fig. S2). The closest relatives to the remaining MAGs were microbes from the same genera isolated from the human body as part of the Human Microbiome Project.

**Figure 6:**
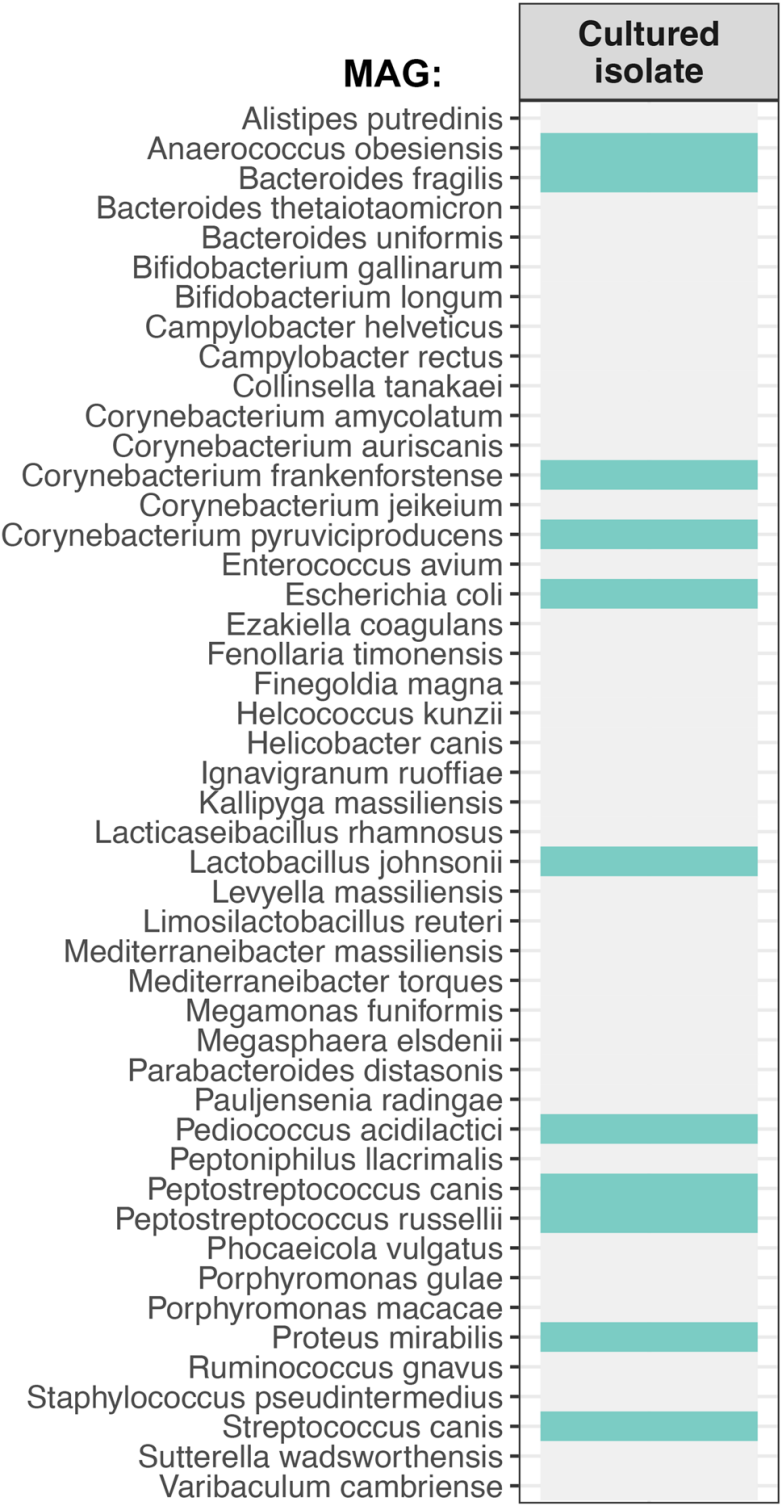
Comparing MAGs and cultured isolates. We investigated whether bacterial species that were represented by one or more MAGs (a total of 47 taxa) were also recovered in the lab as cultured isolates. Turquoise cells indicate that the bacterial taxon was represented by a bacterial isolate, while a grey cell indicates the opposite. It is important to note that MAGs and cultured isolates were assigned taxonomy using different databases.

A total of 11 bacterial species were recovered as MAGs, as cultured isolates and were also present in the larger metagenome dataset (Fig. 6, Table S18). These bacterial species were: *Streptococcus canis*, *Proteus mirabilis*, *Pediococcus acidilactici*, *Lactobacillus johnsonii, Escherichia coli*, *Corynebacterium frankenforstense*, *Bacteroides fragilis*, and *Anaerococcus obesiensis* (Fig. 6). Of these species, the 4 with the highest relative abundances in the microbiome dataset were *C. frankenfortstense* (9.9% mean relative abundance), *P. mirabilis* (7.1%), *L. johnsonii* (6.4%), and *B. fragilis* (5.5%) (Table S8). These four bacterial species make good candidates for further investigation into their potential contributions to fatty acid and volatile compound production.

Analyses showed that MAG profiles varied with host age, which accounted for 8–10% of the variance (PERMANOVA, p < 0.05, Table 2). Furthermore, the anal glands of indoor house cats did not contain the same MAGs as the anal glands of cats that had access to both the indoors and outdoors (PERMANOVA, p < 0.05, Table 2). The remaining predictors, including obesity category, diet, and a medical diagnosis of periodontitis did not significantly predict MAG abundances. MAG profiles were not correlated with metabolite profiles acquired during solid-phase extraction (Mantel Jaccard r = 0.03, p = 0.34; Bray–Curtis r = 0.06, p = 0.17; Aitchison r = 0.05, p = 0.2) or after liquid derivatization (Mantel Jaccard r = 0.05, p = 0.25; Bray–Curtis r = -0.06, p = 0.8; Aitchison r = 0.004, p = 0.44).

### Are anal gland microbiomes distinct from perianal microbiomes?

Because microbiome data from both the anal gland and perianal region were available for six cats, we tested whether the two body-sites harbored different microbiomes. According to PERMANOVA tests, microbiomes from the anal gland did not necessarily contain all of the same microbes that were found in the perianal region (Jaccard index R^2^ = 0.14, p = 0.03), although the two body sites were not distinct when considering the relative abundances of all bacterial taxa (Bray–Curtis R^2^ = 0.10, p = 0.14; Aitchison R^2^ = 0.09 , p = 0.14). There was also evidence of host-specificity in the microbiome, as host identity accounted for 55–60% of the variation (Jaccard R^2^ = 0.55, p = 0.036; Bray–Curtis R^2^ = 0.49, p = 0.13; Aitchison R^2^ = 0.60, p = 0.01). This shows that there is some consistency between the composition of the microbiome in the anal gland and the microbiome in the perianal region within individuals. For plots of microbiome composition from the six cats at the two body sites, see Fig. S3.

The two body-sites did not differ in the abundances of their metagenome-assembled genomes (PERMANOVA Bray–Curtis R^2^ = 0.07, p = 0.35; Aitchison R^2^ = 0.07, p = 0.89); however, they did vary in the MAGs they contained (PERMANOVA Jaccard R^2^ = 0.05, p = 0.006). Lastly, the functions encoded by the perianal and anal gland microbiomes were not fundamentally different at broad levels (Table S19), but were different when examining specific COGs functions and KEGG orthologs (Table S19).

## Discussion

The main purpose of this study was to survey the bacterial, functional, and metabolite composition of the anal gland microbiome in 23 domestic cats in efforts to identify bacterial taxa or gene pathways potentially involved in the synthesis of volatile organic compounds (VOCs) used by the host during chemical signaling. We also add to the limited literature on the anal gland microbiome and metabolome of cats.

### Microbiome composition of the anal gland in felines compared to other scent-producing mammals

Our work demonstrated that the bacterial genera with the highest relative abundances in the anal gland of domestic cats were *Corynebacterium*, *Bacteroides*, *Proteus*, *Lactobacillus*, *Streptococcus*, and *Peptoniphilus*. This is distinct from what has been reported for the anal glands of a Bengal cat (*Felis catus* × *Prionailurus bengalensis*), where 95% of the microbiome community was comprised by *Tessaracoccus*, *Anaerococcus*, and *Finegoldia *^34^. These differences might be attributed to host species differences (domestic cat vs. Bengal cat) or study methodologies (e.g. amplicon sequencing in the Bengal cat study vs. shotgun sequencing here). The anal gland microbiomes of domestic dogs, however, harbored relative abundances of *Bacteroides* and *Proteus* similar to what was found in the surveyed cats^38^. In red foxes, *Proteus mirabilis* is consistently isolated from anal gland secretions^69^. Similar to the cats in this study, the anogenital gland secretions of giant pandas are dominated by *Corynebacterium* (and *Pseudomonas*, and *Porphyromonas*)^35^. The anal gland microbiome of the surveyed cats however did not mirror that found in wild spotted hyenas (*Crocuta crocuta*), where 95% of sequences were classified as *Clostridiales* (*Anaerococcus*, *Clostridium*, *Fastidiosipila*, *Finegoldia*, *Peptoniphilus*, and *Tissierella*)^25^. This could be due to differences between the two studies in terms of their genomic sequencing, or reference databases, or to differences in the diet, habitat, physiology, social behavior, and social structure of the two species.

### Predictors of microbiome variation in the anal gland

The anal gland microbiomes of the domestic cats were differentiated by host age and obesity. The cats in our study were all adults and varied in age from 2 to 14 years old. Age-specific differences in the microbiome could be due to physiological, immunological and behavioral changes experienced by cats as they age^70^. Regarding obesity, no prior studies conducted in other scent-producing mammals have compared the scent gland microbiomes of obese and non-obese individuals. The fecal microbiomes of domestic cats however, do vary between obese and non-obese individuals^71,72^. Systemic health conditions associated with feline obesity, including insulin resistance, urinary disease, and cardiovascular disease^73^—may underlie microbiome changes. Lastly, the anal gland microbiomes of the surveyed felines showcased tremendous amounts of individual variability, echoing what was found for domestic dogs^38^. We encourage future studies to sample more widely and identify the ‘core’ bacteria that are found across cats in a larger dataset.

Interestingly, we did find evidence that the microbiome functional repertoires in the anal gland were associated with host living environment; cats that lived indoors had microbiome functions that were not identical to cats that had outdoor access. It is thought that cats with outdoor access are more likely to be infected with parasites than indoor-only cats^74^ but may have more exposure to natural enrichment and mental stimulation. Conversely, cats that have an indoor-only lifestyle may experience reduced physical activity, greater food consumption, and less natural enrichment^75^. Nonetheless, it is not clear how these lifestyle differences and contact with the outdoors could be linked to functional differences in the anal gland microbiome.

It is important to acknowledge that another factor and source of variation in anal gland microbiome profiles could be the perianal skin surrounding the gland itself and the rectum. Due to their physical proximity and host grooming behaviors, it is possible that there are microbes in the anal gland that came from the skin or rectum.

### Metabolite composition of the anal gland in felines compared to other scent-producing mammals

The classes of metabolites detected in the anal gland of companion cats were consistent with those previously found in the glandular secretions of European badgers^30^, red foxes^76^, meerkats^36^, domestic dogs^77^, coyotes^78^, and giant pandas^35^. Similar to cats, the glandular microbiomes of these mammalian species also contained volatile compounds such as aldehydes, hydrocarbons, fatty acids, ketones, esters, alcohols, or aromatic compounds.

Although the chemical composition of scent gland secretions were not identical between the surveyed cats and Bengal cats^34^, other domestic cats^42^, or domestic dogs^77^, they did overlap in regards to certain compounds. The anal glands of other domestic cats and the cats in this study both contained butanoic acid, methylbutanoic acid, and pentanoic acid^42^. Compared to dogs, the anal glands of cats also contained benzene compounds, phenols, and several fatty acids such as pentanoic, butanoic, and pentadecenoic acids^77^. These acids are also a constituent of fox^79^, pig^80^, and human body odors^81^.

Compounds such as indole, xylene, hexadecanoic acid, and nonanoic acid were detected in the anal glands of Bengal cats^34^ the cats in our study, and giant pandas^35^. However, xylene has not been widely reported in the glands of other canids, mustelids, or primates and its relevance to host chemical communication is unknown. Indole is an aromatic compound produced by the bacterial deamination of the amino acid tryptophan and has been recovered from the skin of reticulated giraffes^82^, and the volatile headspace of human sweat^83^. This compound is an essential metabolite involved in plant–insect interactions but again its significance to odor composition and chemical signaling in mammals has yet to be demonstrated.

### Correlations between microbes and metabolites in the anal gland

Although not all compounds found in the glandular secretions of cats can be associated with host odor and chemical communication, our study did find that the metabolome profiles were overall correlated with microbiome profiles. This finding supports prior work conducted in meerkats^36^, giant pandas^35^, striped and spotted hyenas^25^, and European badgers^30^. In Bengal cats, an even more direct link exists given that bacteria cultured from the anal gland produced many of the same volatiles detected in anal gland secretions^34^. Experimental evidence that microbes directly contribute to chemical signaling is also documented in humans, where microbes isolated from the skin can produce volatile compounds that attract African malaria mosquitos^84^.

In our study, the relative abundances of several anal gland bacteria were positively correlated with the relative abundances of isovaleric acid and nonanoic acid (and several unidentified metabolites). They were bacteria from the genus *Clostridium*, *Streptococcus*, *Fenollaria*, and *Ezakiella.* Isovaleric acid has been recovered from the anal glands of dogs^77^, coyotes^78^, and red foxes^76^. Two enzymes—a transaminase and a decarboxylase—are involved in its synthesis and both have been purified from *Clostridium bifermentans *^85^. Isovaleric acids have also been recovered from cultures of four other *Clostridium* species^86^.

Nonanoic acid was one of six carboxylic acids that impacted the aroma of yogurt fermented by *Streptococcus thermophilus *^87^. These fatty-acids can be produced from the oxidation of nonanal—an aldehyde—via the activity of aldehyde dehydrogenases (ALDH). NADP-dependent ALDH have been characterized in *Streptococcus mutans *^88^, indicating that *S. mutans* and relatives may have the functional capacity to produce nonanoic acid and other carboxylic acids.

The relative abundances of two bacterial species—*Clostridium septicum*, and *Fenollaria sporofastidious*—were negatively correlated with the relative abundances of epicholestanol and cholesterol. Cholesterol is ubiquitous in animal cell membranes, and can be oxidized into cholesterol aldehydes. It has been found in high relative abundances in the anal glands of giant pandas^35^ and alpine marmots^27^. However, its relevance to host odor and chemical communication in cats is not clear.

### Four potentially important bacterial species of the felid anal gland

We call attention to four MAGs that were abundant in the larger microbiome dataset and were recovered as cultured isolates: *Corynebacterium frankenforstense*, *Proteus mirabilis*, *Lactobacillus johnsonii*, and *Bacteroides fragilis*. These four bacterial taxa represent candidates for further study into their contributions to mammalian scent and chemical communication.

Prior studies show that *Corynebacteria* are common inhabitants of the anal glands^39^, perineal glands^89^, musk glands^90^, and axillae^91^ of mammals. Researchers performing both correlative and experimental studies report that *Corynebacteria* can cleave odorant precursors present in the human armpit which leads to the release of short-branched fatty acids that are key components of axillary odor^92^.

*P. mirabilis* isolated from the salivary gland of blow fly maggots secrete indoles, carboxylic acids, and phenols to attract blowflies to animal carcasses^93^. *P. vulgaris*, a close relative of *P. mirabilis*, produces the largest number of aromatic compounds (e.g. esters, ketones, aldehydes, alcohols, and sulfides) during cheese ripening out of any other bacteria present in French cheese rinds^94^. *P. mirabilis* have also been shown to possess fatty acid decarboxylases and alcohol dehydrogenases, which are required for VOC synthesis^95,96^.

*Bacteroides fragilis* isolated from the anal gland of a Bengal cat produced the same volatile compounds present in anal gland secretions^34^. *Bacteroides* spp., which are one of the most abundant gram-negative bacteria in the human gut, are well-known producers of short-chain fatty acids including acetic, isobutyric, propionic, isovaleric, and succinic acids^97^.

Although *Lactobacillus* spp. are not typically found in anal gland secretions (with the exception of European badgers^30^), they can ferment sugars to produce lactate, acetate, or ethanol; the latter two which are anal gland volatile compounds^98^. Lactic acid (the nonaqueous form of lactate) is detected in the perineal glands of North American porcupines^89^ and tarsal glands of white-tailed deer^99^. Furthermore, in the harlequin ladybird beetle, *Lactobacillus* spp. produce volatiles that function as important semiochemicals during host antipredator defense behavior^100^.

### Limitations and future directions

One of the study’s biggest limitation was the potential cross-contamination between anal gland, perianal skin, and rectal bacteria. Due to the location of the anal gland opening and the technique via which anal glands are expressed to extract material, it was physically impossible to avoid contamination of perianal skin-associated microbes. Thus, anal gland microbiomes could contain bacteria that originated from the skin or rectum. To minimize other sources of contamination, we also recommend future studies align shotgun sequences to the human genome, in addition to the host genome.

Another significant limitation of the study was the small sample size. More robust and comprehensive findings can emerge with a larger dataset. Statistically robust correlations between the relative abundances of bacterial species and host characteristics like body condition, diet, or disease would be possible. In the current study, we described associations between bacterial abundances and host characteristics but these were not evaluated statistically and should be interpreted with caution.

As mentioned in our article, the cats in our study were clinically healthy with normal anal glands, but the cats had been previously diagnosed with diseases that affected other areas of the body. This is something to keep in mind for future studies that want to compare their findings to ours. If future investigations elect to focus on disease, they can gather a larger number of cats with and without a specific disease (e.g. lymphoma, diabetes, urinary tract disease) and compare their microbiomes and MAGs. With MAGs in particular, further annotation can be done to characterize the genes present and identify any toxigenic genes or pathogenic strains.

Briefly, we also want to acknowledge technical limitations. The databases used to assign taxonomy to sequences (Kraken DB, GTDB, NCBI RefSeq) have their own set of biases, assumptions, and limitations, all of which unavoidably influenced our results. We used the software’s default parameters, and changing to more stringent parameters will affect the results. Furthermore, changing to another taxonomic classifier tool (e.g. MetaPhlAn 4, Kaiju, Centrifuge) could yield different results as well. We advise readers to keep these limitations in mind as they compare our findings to theirs.

Despite these limitations, our study fills in a large gap in the literature as only few studies have examined the microbes or metabolites in the anal glands of cats. We provide a combined analysis of the felid microbiome and metabolome in the anal gland and share metagenome-assembled genomes (MAGs) for this body site for this species which future work can build upon.

### Supplementary Information

Supplementary Figures.

Supplementary Tables.

## Data Availability

Raw shotgun sequences have been uploaded to the NCBI Sequence Read Archive (SRA) (accession numbers SRR24332691-SRR24332721), and fastq files from the 85 MAGs were deposited into NCBI Genomes (accessions JASBWX000000000-JASCAD000000000, Table S4). Both types of genetic data are housed under the BioProject PRJNA961122. The ASV relative abundance table, ASV taxonomic classifications, and corresponding sample metadata are available as supplementary materials. The R code for conducting all statistical analyses and generating all figures presented in this article is stored in a public GitHub repository (https://github.com/rojascon/Cat_AnalGland_Microbiome_Metabolome).

## References

[1] Albone, E. S. Mammalian semiochemistry. The investigation of chemical signals between mammals. 2–5 (1984).

[2] Wyatt TD (2003). Pheromones and Animal Behaviour: Communication by Smell and Taste.

[3] Doty RL (1986). Odor-guided behavior in mammals. Experientia.

[4] Brennan PA, Zufall F (2006). Pheromonal communication in vertebrates. Nature..

[5] Colquhoun IC (2011). A review and interspecific comparison of nocturnal and cathemeral strepsirhine primate olfactory behavioural ecology. Int. J. Zool..

[6] delBarco-Trillo J, Drea CM (2014). Socioecological and phylogenetic patterns in the chemical signals of strepsirrhine primates. Anim. Behav..

[7] Janssenswillen S, Roelants K, Carpentier S, de Rooster H, Metzemaekers M, Vanschoenwinkel B (2021). Odorant-binding proteins in canine anal sac glands indicate an evolutionarily conserved role in mammalian chemical communication. BMC Ecol. Evol..

[8] Burger BV, Schulz S (2005). Mammalian semiochemicals. The Chemistry of Pheromones and Other Semiochemicals II.

[9] de Lacy CB, Amann A, Al-Kateb H, Flynn C, Filipiak W, Khalid T (2014). A review of the volatiles from the healthy human body. J. Breath Res..

[10] Gustin MK, McCracken GF (1987). Scent recognition between females and pups in the bat *Tadarida brasiliensis* mexicana. Anim. Behav..

[11] Porter RH, Moore JD (1981). Human kin recognition by olfactory cues. Physiol Behav..

[12] Henkel S, Setchell JM (2018). Group and kin recognition via olfactory cues in chimpanzees (*Pan troglodytes*). Proc. Biol. Sci..

[13] Drea CM, Vignieri SN, Kim HS, Weldele ML, Glickman SE (2002). Responses to olfactory stimuli in spotted hyenas (Crocuta crocuta): II. Discrimination of conspecific scent. J. Comp. Psychol..

[14] Asa CS, Mech LD, Seal US (1985). The use of urine, faeces, and anal-gland secretions in scent-marking by a captive wolf (*Canis lupus*) pack. Anim. Behav..

[15] Adams DM, Li Y, Wilkinson GS (2018). Male scent gland signals mating status in greater spear-nosed bats, *Phyllostomus*
*hastatus*. J. Chem. Ecol..

[16] Fan M, Zhang M, Shi M, Zhang T, Qi L, Yu J (2018). Sex hormones play roles in determining musk composition during the early stages of musk secretion by musk deer (*Moschus **berezovskii*). Endocr. J..

[17] Heymann EW (2006). Scent marking strategies of New World primates. Am. J. Primatol..

[18] Voigt CC, Behr O, Caspers B, von Helversen O, Knörnschild M, Mayer F (2008). Songs, scents, and senses: Sexual selection in the greater sac-winged bat, *Saccopteryx*
*bilineata*. J. Mammal..

[19] Begg CM, Begg KS, Du Toit JT, Mills MGL (2003). Scent-marking behaviour of the honey badger, *Mellivora capensis* (Mustelidae), in the southern Kalahari. Anim. Behav..

[20] Wood WF, Sollers BG, Dragoo GA, Dragoo JW (2002). Volatile components in defensive spray of the hooded skunk, *Mephitis macroura*. J. Chem. Ecol..

[21] Poirotte C, Massol F, Herbert A, Willaume E, Bomo PM, Kappeler PM (2017). Mandrills use olfaction to socially avoid parasitized conspecifics. Sci. Adv..

[22] Rosell F, Kniha D, Haviar M (2020). Dogs can scent-match individual Eurasian beavers from their anal gland secretion. Wildl. Biol..

[23] Kücklich M, Weiß BM, Birkemeyer C, Einspanier A, Widdig A (2019). Chemical cues of female fertility states in a non-human primate. Sci. Rep..

[24] Miyazaki M, Miyazaki T, Nishimura T, Hojo W, Yamashita T (2018). The chemical basis of species, sex, and individual recognition using feces in the domestic cat. J. Chem. Ecol..

[25] Theis KR, Venkataraman A, Dycus JA, Koonter KD, Schmitt-Matzen EN, Wagner AP (2013). Symbiotic bacteria appear to mediate hyena social odors. Proc. Natl. Acad. Sci. USA..

[26] Bornbusch, S. L. *et al.* Stable and transient structural variation in lemur vaginal, labial and axillary microbiomes: Patterns by species, body site, ovarian hormones and forest access. *FEMS Microbiol. Ecol.* **96**. 10.1093/femsec/fiaa090 (2020). 10.1093/femsec/fiaa090 32401310

[27] Zidat T, Dufour A-B, Meiffren G, Gabirot M, Comte G, Allainé D (2018). Anal scent gland secretions inform on sexual maturity, sex and social status in the Alpine marmot, *Marmota marmota* (Rodentia: Sciuridae): A role in intrasexual competition in cooperative breeders?. Biol. J. Linn. Soc. Lond..

[28] Henkel S, Lambides AR, Berger A, Thomsen R, Widdig A (2015). Rhesus macaques (*Macaca mulatta*) recognize group membership via olfactory cues alone. Behav. Ecol. Sociobiol..

[29] Noonan MJ, Tinnesand HV, Müller CT, Rosell F, Macdonald DW, Buesching CD (2019). Knowing me, knowing you: Anal gland secretion of European badgers (*Meles meles*) codes for individuality, sex and social group membership. J. Chem. Ecol..

[30] Buesching, C. D., Tinnesand, H. V., Sin, Y., Rosell, F., Burke, T. & Macdonald, D. W. Coding of group odor in the subcaudal gland secretion of the European badger *Meles meles*: chemical composition and pouch microbiota. Chemical signals in vertebrates, vol. 13. 45–62 (Springer International Publishing, 2016).

[31] Pelosi P, Knoll W (2022). Odorant-binding proteins of mammals. Biol. Rev. Camb. Philos. Soc..

[32] Shortall K, Djeghader A, Magner E, Soulimane T (2021). Insights into aldehyde dehydrogenase enzymes: A structural perspective. Front. Mol. Biosci..

[33] Paiva P, Medina FE, Viegas M, Ferreira P, Neves RPP, Sousa JPM (2021). Animal fatty acid synthase: A chemical nanofactory. Chem. Rev..

[34] Yamaguchi MS, Ganz HH, Cho AW, Zaw TH, Jospin G, McCartney MM (2019). Bacteria isolated from Bengal cat (*Felis catus* × *Prionailurus bengalensis*) anal sac secretions produce volatile compounds potentially associated with animal signaling. PLoS ONE..

[35] Zhou W, Qi D, Swaisgood RR, Wang L, Jin Y, Wu Q (2021). Symbiotic bacteria mediate volatile chemical signal synthesis in a large solitary mammal species. ISME J..

[36] Leclaire S, Jacob S, Greene LK, Dubay GR, Drea CM (2017). Social odours covary with bacterial community in the anal secretions of wild meerkats. Sci. Rep..

[37] Netzker T, Shepherdson EMF, Zambri MP, Elliot MA (2020). Bacterial volatile compounds: Functions in communication, cooperation, and competition. Annu. Rev. Microbiol..

[38] Bergeron CC, Costa MC, de Souza LB, Sauvé F (2021). Description of the bacterial microbiota of anal sacs in healthy dogs. Can. J. Vet. Res..

[39] Theis KR, Schmidt TM, Holekamp KE (2012). Evidence for a bacterial mechanism for group-specific social odors among hyenas. Sci. Rep..

[40] Martín-Vivaldi M, Peña A, Peralta-Sánchez JM, Sánchez L, Ananou S, Ruiz-Rodríguez M (2010). Antimicrobial chemicals in hoopoe preen secretions are produced by symbiotic bacteria. Proc. Biol. Sci..

[41] Whittaker, D. J. *et al.* Experimental evidence that symbiotic bacteria produce chemical cues in a songbird. *J. Exp. Biol.* **222**. 10.1242/jeb.202978 (2019). 10.1242/jeb.202978 31537652

[42] Miyazaki T, Nishimura T, Yamashita T, Miyazaki M (2018). Olfactory discrimination of anal sac secretions in the domestic cat and the chemical profiles of the volatile compounds. J. Ethol..

[43] Cole JR, Wang Q, Fish JA, Chai B, McGarrell DM, Sun Y (2014). Ribosomal Database Project: Data and tools for high throughput rRNA analysis. Nucleic Acids Res..

[44] O’Leary NA, Wright MW, Brister JR, Ciufo S, Haddad D, McVeigh R (2016). Reference sequence (RefSeq) database at NCBI: Current status, taxonomic expansion, and functional annotation. Nucleic Acids Res..

[45] Bolger AM, Lohse M, Usadel B (2014). Trimmomatic: A flexible trimmer for Illumina sequence data. Bioinformatics..

[46] Langmead B, Salzberg SL (2012). Fast gapped-read alignment with Bowtie 2. Nat. Methods..

[47] Wood DE, Lu J, Langmead B (2019). Improved metagenomic analysis with Kraken 2. Genome Biol..

[48] Lu J, Breitwieser FP, Thielen P, Salzberg SL (2017). Bracken: Estimating species abundance in metagenomics data. PeerJ Comput. Sci..

[49] Boisvert S, Laviolette F, Corbeil J (2010). Ray: Simultaneous assembly of reads from a mix of high-throughput sequencing technologies. J. Comput. Biol..

[50] Nurk S, Meleshko D, Korobeynikov A, Pevzner PA (2017). metaSPAdes: A new versatile metagenomic assembler. Genome Res..

[51] Gurevich A, Saveliev V, Vyahhi N, Tesler G (2013). QUAST: Quality assessment tool for genome assemblies. Bioinformatics..

[52] Eren AM, Kiefl E, Shaiber A, Veseli I, Miller SE, Schechter MS (2021). Community-led, integrated, reproducible multi-omics with anvi’o. Nat. Microbiol..

[53] Hyatt D, Chen G-L, Locascio PF, Land ML, Larimer FW, Hauser LJ (2010). Prodigal: Prokaryotic gene recognition and translation initiation site identification. BMC Bioinform..

[54] Galperin MY, Wolf YI, Makarova KS, Vera Alvarez R, Landsman D, Koonin EV (2021). COG database update: Focus on microbial diversity, model organisms, and widespread pathogens. Nucleic Acids Res..

[55] Kanehisa M, Furumichi M, Sato Y, Kawashima M, Ishiguro-Watanabe M (2023). KEGG for taxonomy-based analysis of pathways and genomes. Nucleic Acids Res..

[56] Patro R, Duggal G, Love MI, Irizarry RA, Kingsford C (2017). Salmon provides fast and bias-aware quantification of transcript expression. Nat. Methods..

[57] Kang DD, Li F, Kirton E, Thomas A, Egan R, An H (2019). MetaBAT 2: An adaptive binning algorithm for robust and efficient genome reconstruction from metagenome assemblies. PeerJ..

[58] Parks DH, Imelfort M, Skennerton CT, Hugenholtz P, Tyson GW (2015). CheckM: Assessing the quality of microbial genomes recovered from isolates, single cells, and metagenomes. Genome Res..

[59] Chaumeil P-A, Mussig AJ, Hugenholtz P, Parks DH (2019). GTDB-Tk: A toolkit to classify genomes with the Genome Taxonomy Database. Bioinformatics..

[60] Parks DH, Chuvochina M, Chaumeil P-A, Rinke C, Mussig AJ, Hugenholtz P (2020). A complete domain-to-species taxonomy for Bacteria and Archaea. Nat. Biotechnol..

[61] Stamatakis A (2014). RAxML version 8: A tool for phylogenetic analysis and post-analysis of large phylogenies. Bioinformatics..

[62] Callahan BJ, McMurdie PJ, Rosen MJ, Han AW, Johnson AJA, Holmes SP (2016). DADA2: High-resolution sample inference from Illumina amplicon data. Nat Methods..

[63] R Core Team. *R: A Language and Environment for Statistical Computing* (R Foundation for Statistical Computing, 2021). https://www.R-project.org.

[64] Davis NM, Proctor DM, Holmes SP, Relman DA, Callahan BJ (2018). Simple statistical identification and removal of contaminant sequences in marker-gene and metagenomics data. Microbiome..

[65] Wickham H (2009). ggplot2: Elegant Graphics for Data Analysis.

[66] Teng KT, McGreevy PD, Toribio JALML, Raubenheimer D, Kendall K, Dhand NK (2018). Associations of body condition score with health conditions related to overweight and obesity in cats. J. Small Anim. Pract..

[67] Oksanen, J. vegan : Community Ecology Package. R package version 1.8-5. http://www.cran.r-project.org (2007) (Accessed 11 Oct 2022) https://ci.nii.ac.jp/naid/10020010631/.

[68] van den Berg RA, Hoefsloot HCJ, Westerhuis JA, Smilde AK, van der Werf MJ (2006). Centering, scaling, and transformations: Improving the biological information content of metabolomics data. BMC Genomics..

[69] Gosden PE, Ware GC (1976). The aerobic bacterial flora of the anal sac of the red fox. J. Appl. Bacteriol..

[70] Bellows J, Center S, Daristotle L, Estrada AH, Flickinger EA, Horwitz DF (2016). Aging in cats: Common physical and functional changes. J. Feline Med. Surg..

[71] Ma X, Brinker E, Graff EC, Cao W, Gross AL, Johnson AK (2022). Whole-genome shotgun metagenomic sequencing reveals distinct gut microbiome signatures of obese cats. Microbiol. Spectr..

[72] Kieler IN, Mølbak L, Hansen LL, Hermann-Bank ML, Bjornvad CR (2016). Overweight and the feline gut microbiome—A pilot study. J. Anim. Physiol. Anim. Nutr..

[73] Chiang C-F, Villaverde C, Chang W-C, Fascetti AJ, Larsen JA (2022). Prevalence, risk factors, and disease associations of overweight and obesity in cats that visited the Veterinary Medical Teaching Hospital at the University of California, Davis from January 2006 to December 2015. Top. Companion Anim. Med..

[74] Chalkowski K, Wilson AE, Lepczyk CA, Zohdy S (2019). Who let the cats out? A global meta-analysis on risk of parasitic infection in indoor versus outdoor domestic cats (*Felis catus*). Biol. Lett..

[75] Tan, S. M. L., Stellato, A. C. & Niel, L. Uncontrolled outdoor access for cats: An assessment of risks and benefits. *Animals* **10**. 10.3390/ani10020258 (2020). 10.3390/ani10020258 PMC7070728 32041155

[76] Albone ES, Perry GC (1976). Anal sac secretion of the red fox, *Vulpes vulpes*; volatile fatty acids and diamines: Implications for a fermentation hypothesis of chemical recognition. J. Chem. Ecol..

[77] Dorrigiv I, Hadian M, Bahram M (2023). Comparison of volatile compounds of anal sac secretions between the sexes of domestic dog (*Canis lupus familiaris*). Vet. Res. Forum..

[78] Preti G, Muetterties EL, Furman JM, Kennelly JJ, Johns BE (1976). Volatile constituents of dog (*Canis familiaris*) and coyote (*Canis latrans*) anal sacs. J. Chem. Ecol..

[79] Heale VR, Vanderwolf CH, Kavaliers M (1994). Components of weasel and fox odors elicit fast wave bursts in the dentate gyrus of rats. Behav. Brain Res..

[80] Sheridan BA, Curran TP, Dodd VA (2003). Biofiltration of n-butyric acid for the control of odour. Bioresour. Technol..

[81] Pandey SK, Kim K-H (2011). Human body-odor components and their determination. Trends Analyt. Chem..

[82] Wood WF, Weldon PJ (2002). The scent of the reticulated giraffe (*Giraffa camelopardalis reticulata*). Biochem. Syst. Ecol..

[83] Meijerink J, Braks MAH, Brack AA, Adam W, Dekker T, Posthumus MA (2000). Identification of olfactory stimulants for *Anopheles gambiae* from human sweat samples. J. Chem. Ecol..

[84] Verhulst NO, Beijleveld H, Knols BG, Takken W, Schraa G, Bouwmeester HJ (2009). Cultured skin microbiota attracts malaria mosquitoes. Malar J..

[85] Britz ML, Wilkinson RG (1983). Partial purification and characterization of two enzymes involved in isovaleric acid synthesis in *Clostridium bifermentans*. J. Gen. Microbiol..

[86] Lewis VJ, Moss CW, Jones WL (1967). Determination of volatile acid production of Clostridium by gas chromatography. Can. J. Microbiol..

[87] Zhang L, Mi S, Liu R-B, Sang Y-X, Wang X-H (2020). Evaluation of volatile compounds during the fermentation process of yogurts by *Streptococcus thermophilus* based on odor activity value and heat map analysis. Int. J. Anal. Chem..

[88] Cobessi D, Tête-Favier F, Marchal S, Azza S, Branlant G, Aubry A (1999). Apo and holo crystal structures of an NADP-dependent aldehyde dehydrogenase from *Streptococcus mutans*. J. Mol. Biol..

[89] Roze U, Leung KT, Nix E, Burton G, Chapman DM (2010). Microanatomy and bacterial flora of the perineal glands of the North American porcupine. Can. J. Zool..

[90] Li D, Chen B, Zhang L, Gaur U, Ma T, Jie H (2016). The musk chemical composition and microbiota of Chinese forest musk deer males. Sci Rep..

[91] Callewaert C, Kerckhof F-M, Granitsiotis MS, Van Gele M, Van de Wiele T, Boon N (2013). Characterization of Staphylococcus and Corynebacterium clusters in the human axillary region. PLoS ONE..

[92] Natsch A, Gfeller H, Gygax P, Schmid J, Acuna G (2003). A specific bacterial aminoacylase cleaves odorant precursors secreted in the human axilla. J. Biol. Chem..

[93] Ma Q, Fonseca A, Liu W, Fields AT, Pimsler ML, Spindola AF (2012). *Proteus mirabilis* interkingdom swarming signals attract blow flies. ISME J..

[94] Deetae P, Bonnarme P, Spinnler HE, Helinck S (2007). Production of volatile aroma compounds by bacterial strains isolated from different surface-ripened French cheeses. Appl. Microbiol. Biotechnol..

[95] Wang B, Bai Y, Fan T, Zheng X, Cai Y (2017). Characterisation of a thiamine diphosphate-dependent alpha-keto acid decarboxylase from *Proteus mirabilis* JN458. Food Chem..

[96] Yu F, Bai Y, Fan T-P, Zheng X, Cai Y (2019). Alcohol dehydrogenases from *Proteus mirabilis* contribute to alcoholic flavor. J. Sci. Food Agric..

[97] Sun, X.-W. *et al.**Bacteroides **propionicigenes* sp. nov., isolated from human faeces. *Int. J. Syst. Evol. Microbiol.* **72**. 10.1099/ijsem.0.005397 (2022). 10.1099/ijsem.0.005397 35635547

[98] Hatti-Kaul R, Chen L, Dishisha T, Enshasy HE (2018). Lactic acid bacteria: From starter cultures to producers of chemicals. FEMS Microbiol. Lett..

[99] Alexy KJ, Gassett JW, Osborn DA, Miller KV, Russell SM (2003). Bacterial fauna of the tarsal tufts of white-tailed deer (*Odocoileus virginianus*). Amid..

[100] Schmidtberg H, Shukla SP, Halitschke R, Vogel H, Vilcinskas A (2019). Symbiont-mediated chemical defense in the invasive ladybird *Harmonia **axyridis*. Ecol. Evol..

