# Supplementary material for: Characterization of the microbiome and volatile compounds in anal gland secretions from domestic cats (Felis catus) using metagenomics and metabolomics
Source: Sci Rep. 2023 Nov 8;13:19382. doi: 10.1038/s41598-023-45997-1 (PMC10632438; doi:10.1038/s41598-023-45997-1)

**SUPPLEMENTARY FIGURES FOR:**

**Characterization of the microbiome and metabolome in anal gland secretions from domestic cats (*Felis catus*) using metagenomics and metabolomics**

Connie A. Rojas^1,2*^, Stanley L. Marks^3^, Eva Borras^4,5^, Hira Lesea^6^, Mitchell M. McCartney^4,5,7^, David Coil^1^, Cristina E. Davis^4,5,7^, Jonathan A. Eisen^1,2^

^1^ Genome Center, University of California – Davis, Davis, California, USA

^2^ Department of Evolution and Ecology, University of California – Davis, Davis, California, USA

^3^ Department of Medicine and Epidemiology, University of California – Davis, School of Veterinary Medicine, Davis, California, USA

^4^ Department of Mechanical and Aerospace Engineering, University of California – Davis, Davis, California, USA

^5^ UC Davis Lung Center, University of California – Davis, Davis, CA, USA.

^6^ Department of Microbiology and Molecular Genetics, University of California – Davis, Davis, California, USA

^7^ VA Northern California Health Care System, Mather, CA, USA

**Email the corresponding author with any questions:** [carojas@ucdavis.edu](mailto:carojas@ucdavis.edu)

**List of Figures in this document**

Fig S1 - phylogenetic tree built from MAGs

Fig S2 - phylogenetic trees of six MAGs and their closest relatives in the Genome Taxonomy Database (GTDB)

Fig S3 - plots of microbiome composition in the anal gland and in the perianal region

**Fig S1. Phylogeny of MAGs constructed with alignments from GTDB, rooted to *Fretibacterium*, the only member of the phylum *Synergistota*.**

**
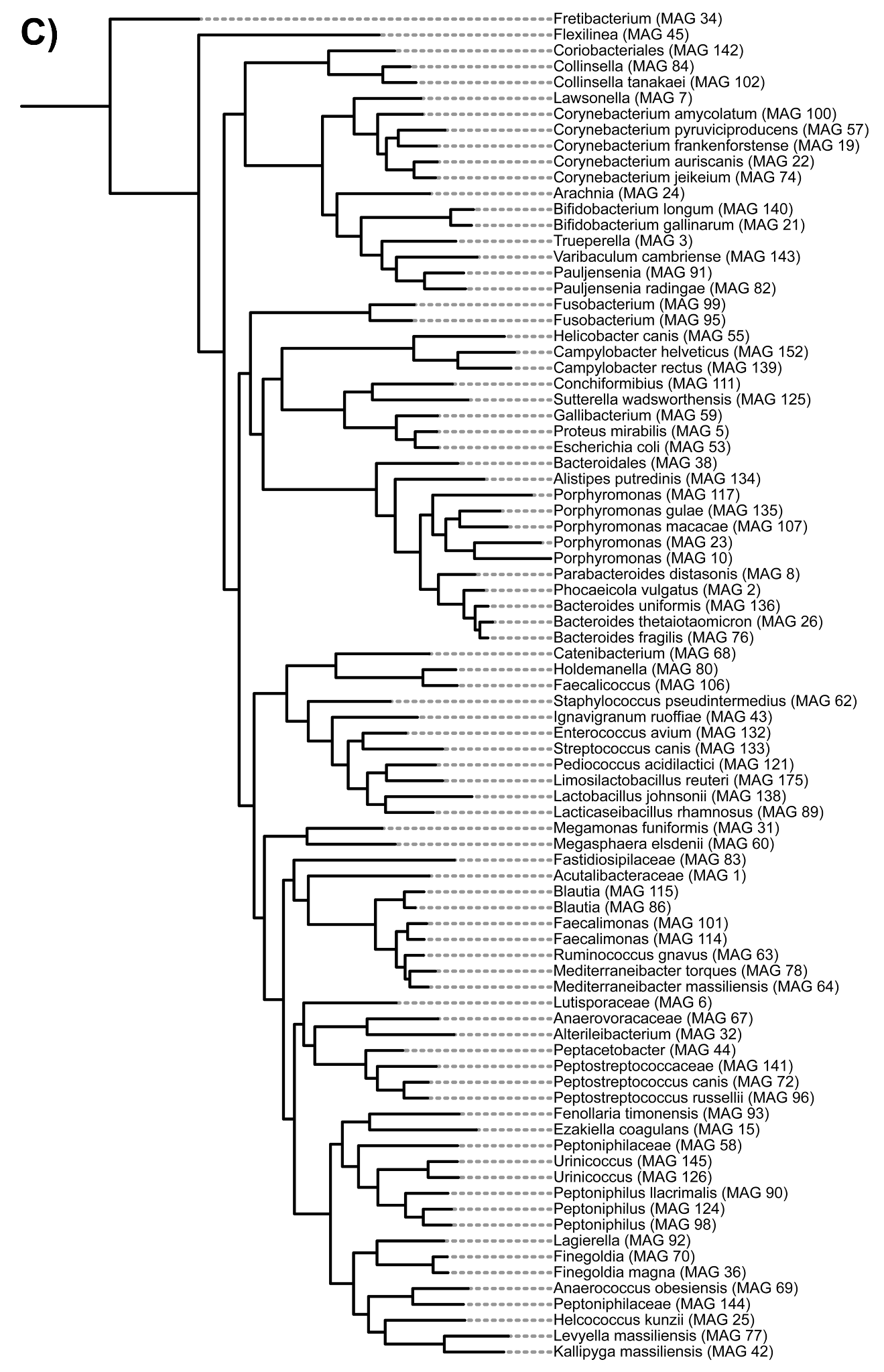
**

**Fig S2. Individual phylogenetic trees of the six most abundant MAGs in the dataset, alongside genomes from the Genome Taxonomy Database (GTDB).** Trees show the evolutionary distance of each MAG to the closest genomes in GTDB, release 202. Nodes of MAGs that came from this study have a purple label.

**MAG #144 *Peptoniphilaceae***

**
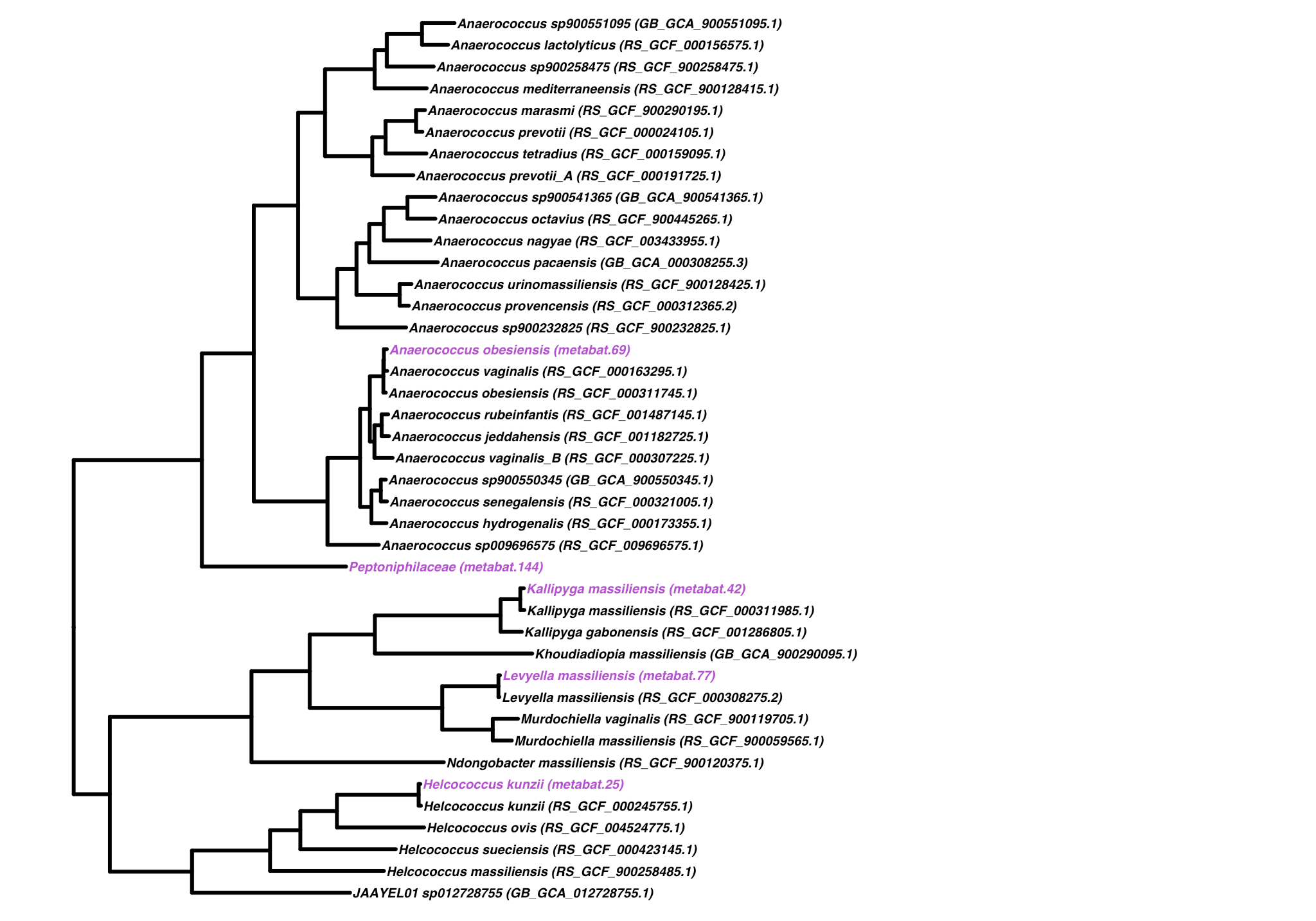
**

**MAG #19 *Corynebacterium* *frankenforstense***

***
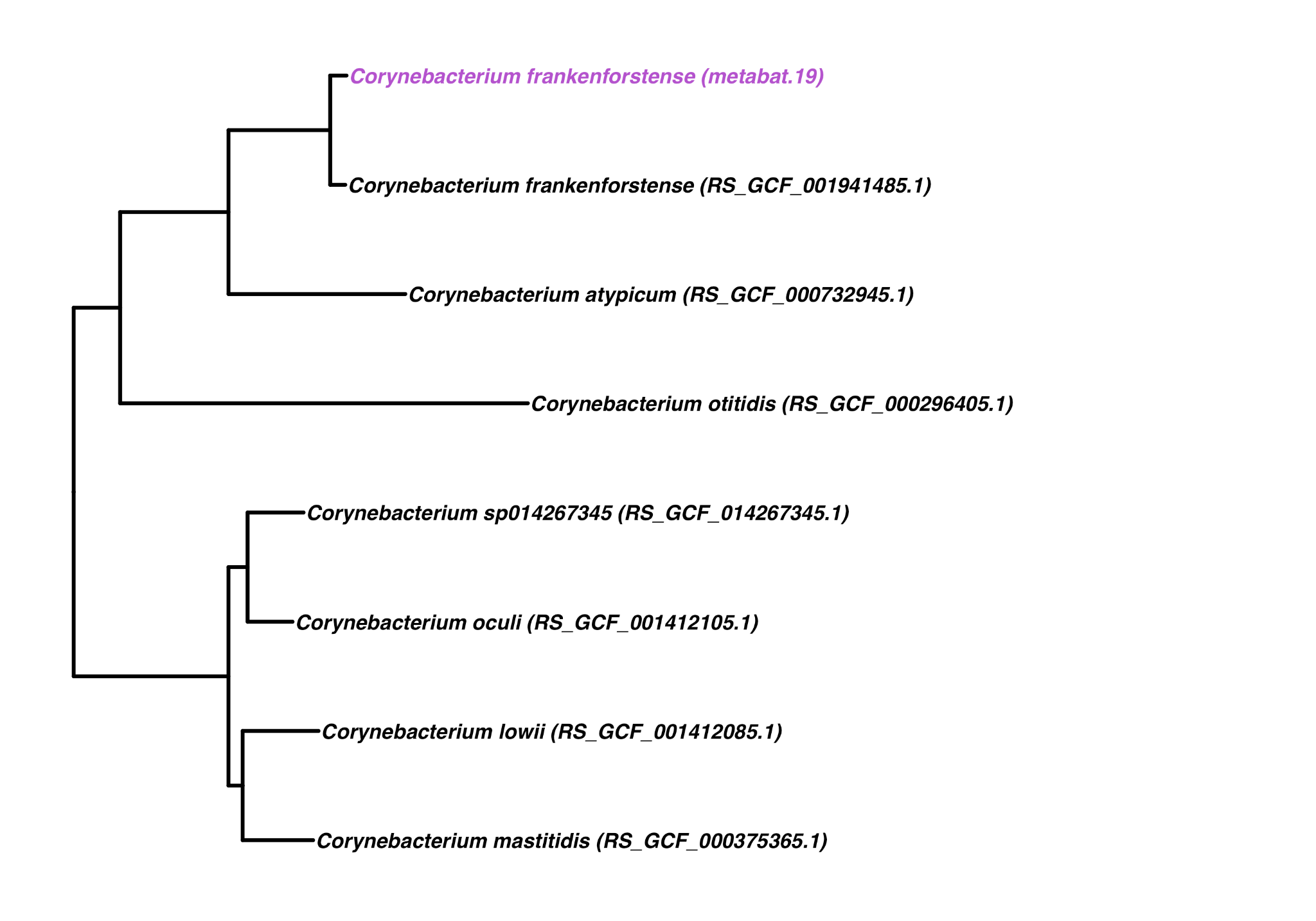
***

**MAG #124 *Peptoniphilus***

**
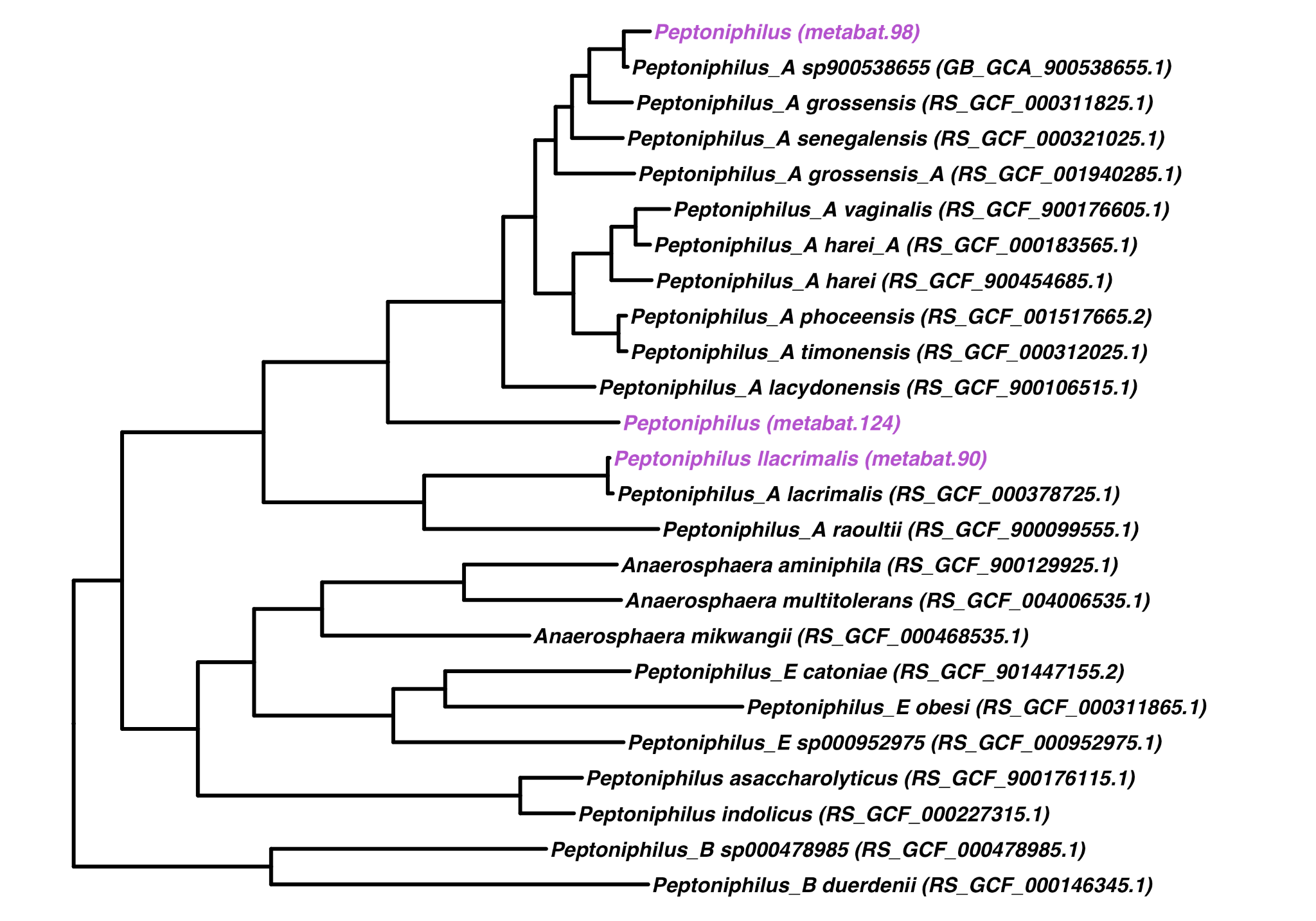
**

**MAG #138 *Lactobacillus johnsonii***

**
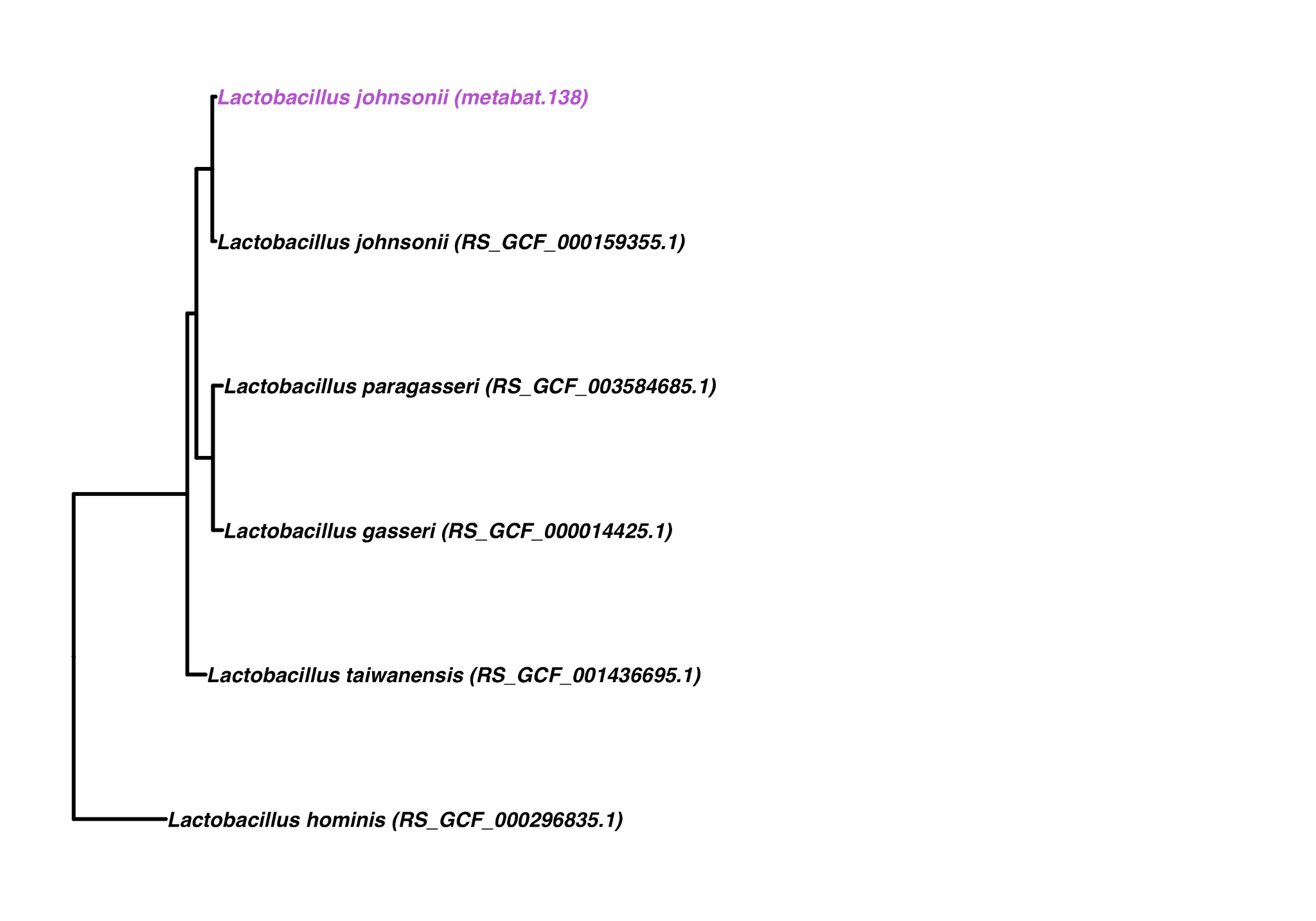
**

**MAG #57 *Corynebacterium pyruviciproducens***

**
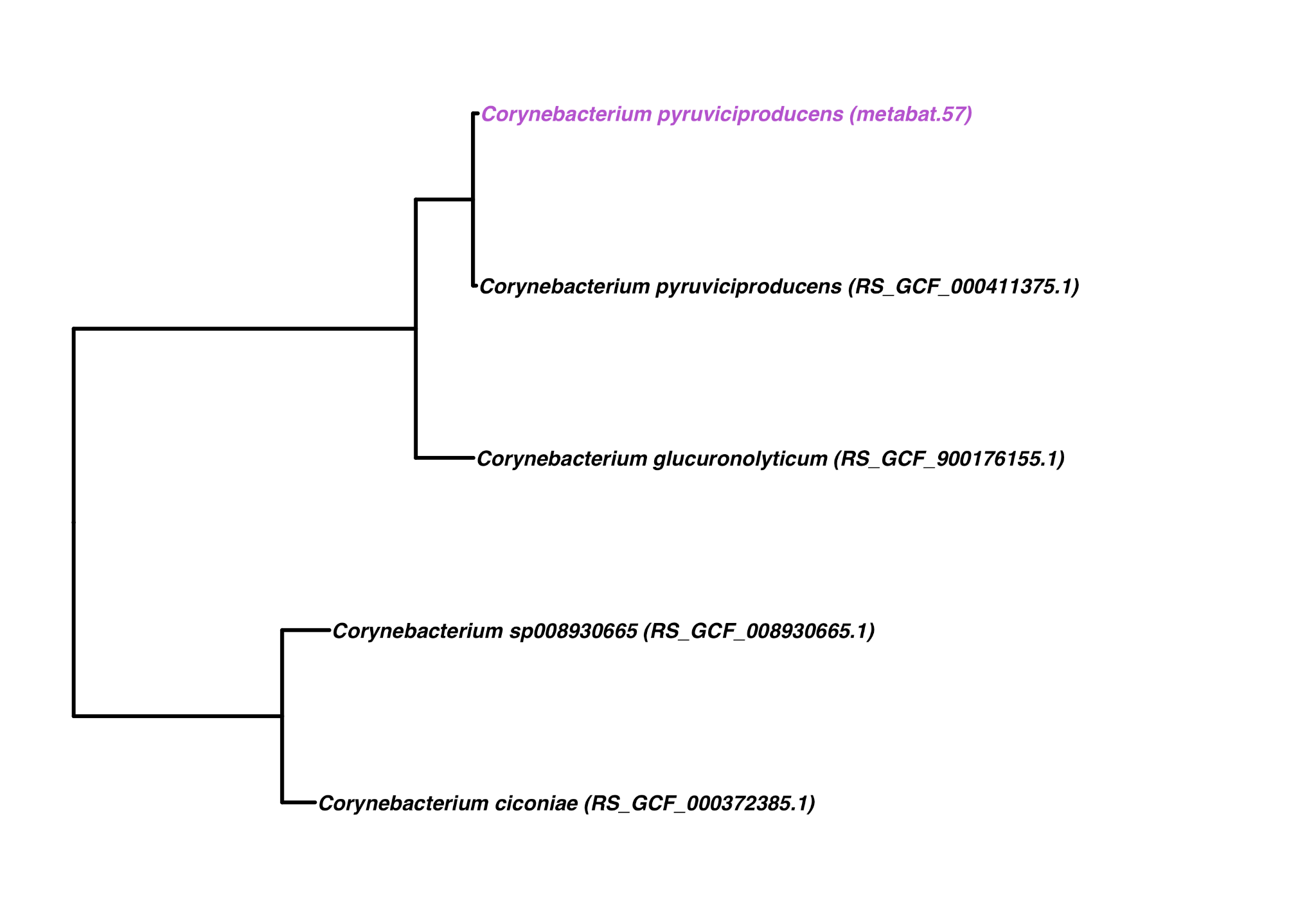
**

**MAG #5 *Proteus mirabilis***

***
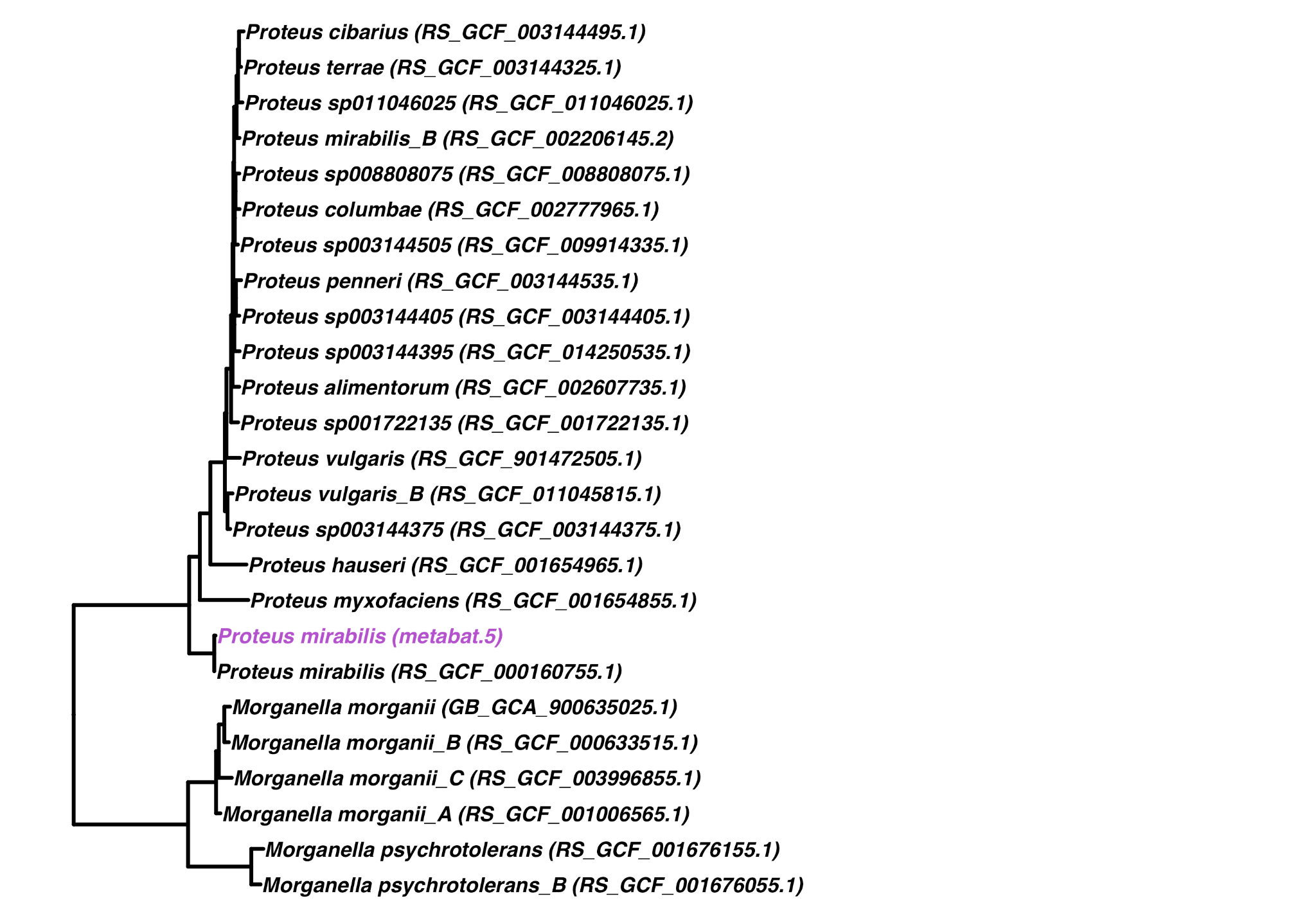
***

**Fig S3. Microbiomes from the anal gland alongside those from the perianal area.** Relative abundances of bacterial genera in the anal gland (left) and perianal region (right) for six feline participants, as estimated from shotgun Illumina sequence data using Kraken2/Bracken. (**Top panels - this page)** Bacterial genera with a mean relative abundance >1.75%. (**Bottom panels - next page**) Bacterial genera with a mean relative abundance >0.38 but <0.76.


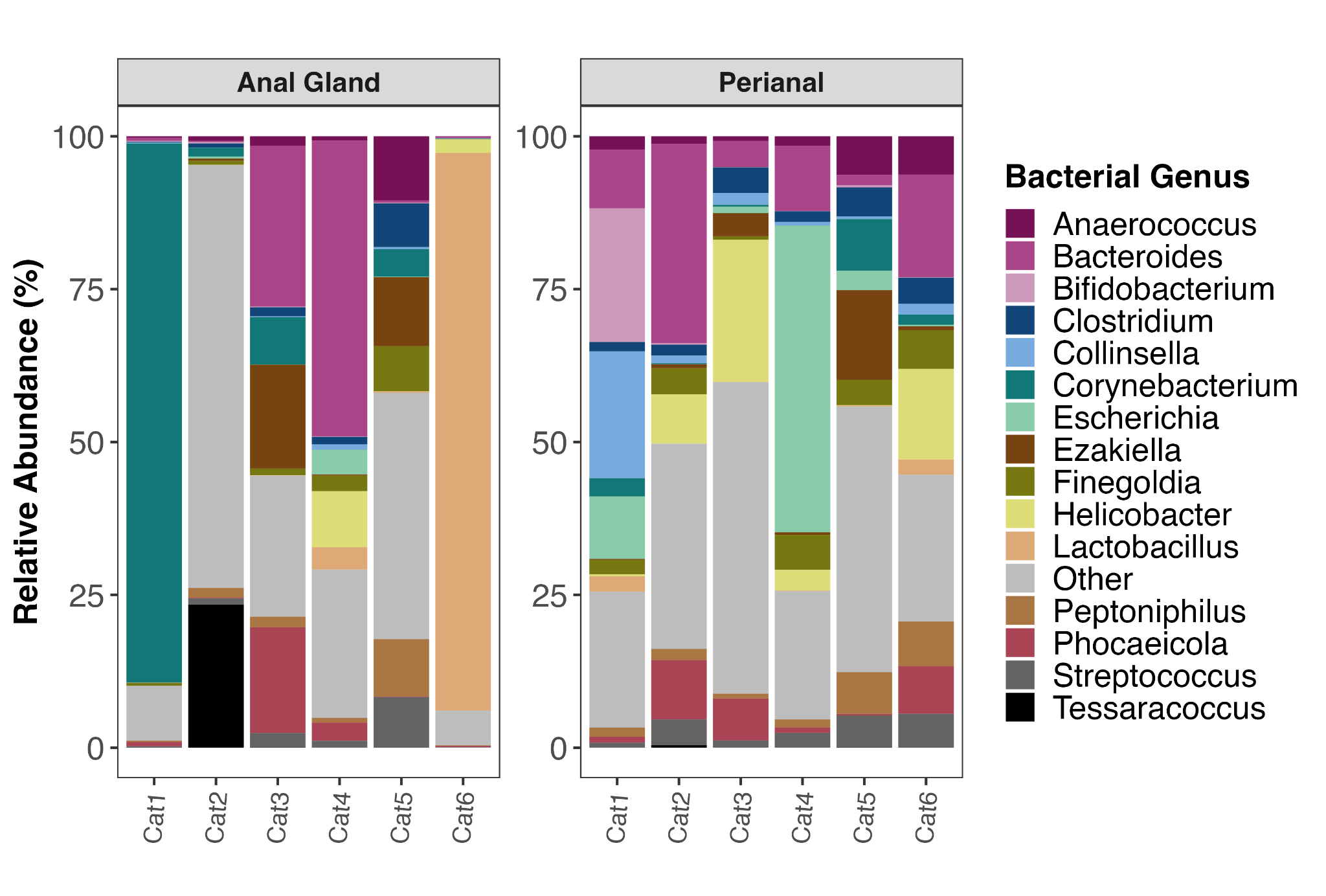


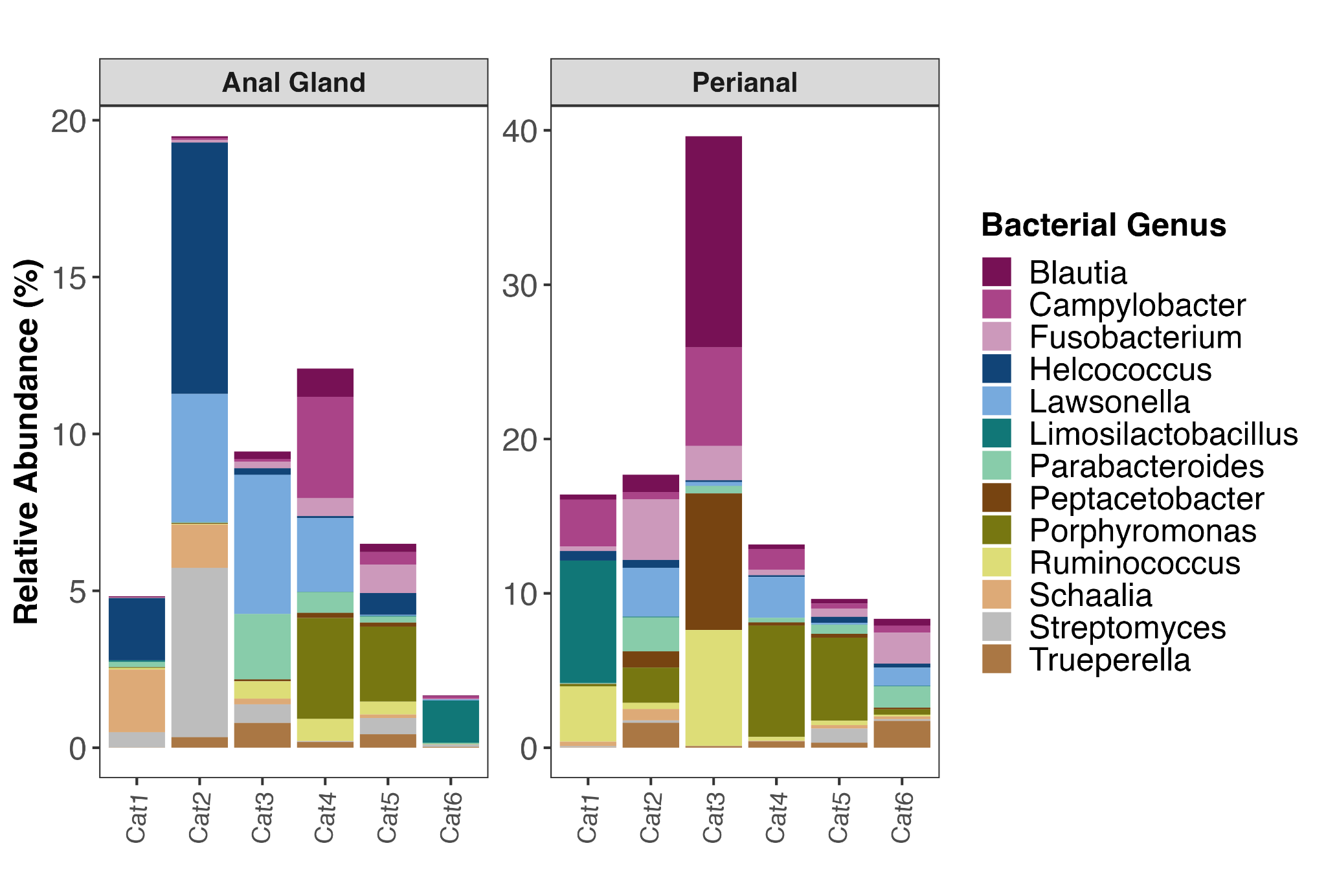

Supplement: Supplementary file 1 — Supplementary Figures. [file 41598_2023_45997_MOESM1_ESM.docx]
